# Actin-like protein 6A/MYC/CDK2 axis confers high proliferative activity in triple-negative breast cancer

**DOI:** 10.1186/s13046-021-01856-3

**Published:** 2021-02-04

**Authors:** Yunting Jian, Xinjian Huang, Lishan Fang, Meng Wang, Qinghua Liu, Hongyi Xu, Lingzhi Kong, Xiangfu Chen, Ying Ouyang, Xi Wang, Weidong Wei, Libing Song

**Affiliations:** 1 Department of Experimental Research, Sun Yat-sen University Cancer Center, State Key Laboratory of Oncology in South China, Collaborative Innovation Center for Cancer Medicine, Guangzhou, 510060 China; 2 Central Laboratory, The Eighth Affiliated Hospital, Sun Yat-sen University, No.3025 Shennan Load, Shenzhen, Guangdong 518033 P.R. China; 3 Department of Breast Surgery, State Key Laboratory of Oncology in Southern China, Collaborative Innovation Center for Cancer Medicine, Sun Yat-sen University Cancer Center, Guangzhou, China

**Keywords:** TNBC, Proliferation, ACTL6A, MYC, CDK2 inhibitor

## Abstract

**Background:**

Triple negative breast cancer (TNBC) is an aggressive subtype of breast cancer with high proliferative activity. TNBC tumors exhibit elevated MYC expression and altered expression of MYC regulatory genes, which are associated with tumor progression and poor prognosis; however, the underlying mechanisms by which MYC retains its high expression and mediates TNBC tumorigenesis require further exploration.

**Methods:**

ACTL6A regulation of MYC and its target gene, CDK2, was defined using Co-IP, mass spectrometry and ChIP assays. To study the role of ACTL6A in TNBC, we performed soft-agar, colony formation, flow cytometry and tumor formation in nude mice. CDK2 inhibitor and paclitaxel were used in testing combination therapy in vitro and in vivo.

**Results:**

ACTL6A bound MYC to suppress glycogen synthase kinase 3 beta (GSK3β)-induced phosphorylation on MYC T58, which inhibited ubiquitination of MYC and stabilized it. Moreover, ACTL6A promoted the recruitment of MYC and histone acetyltransferase KAT5 on CDK2 promoters, leading to hyperactivation of CDK2 transcription. ACTL6A overexpression promoted, while silencing ACTL6A suppressed cell proliferation and tumor growth in TNBC cells in vitro and in vivo*,* which was dependent on MYC signaling. Furthermore, co-therapy with paclitaxel and CDK2 inhibitor showed synergistic effects in tumor suppression. Notably, ACTL6A/MYC/CDK2 axis was specifically up-regulated in TNBC and high expression of ACTL6A was correlated to shorter survival in patients with TNBC.

**Conclusions:**

These findings reveal a novel mechanism by which ACTL6A prolongs the retention of MYC in TNBC and suggest that pharmacological targeting ACTL6A/MYC/CDK2 axis might have therapeutic potential in patients with TNBC.

**Supplementary Information:**

The online version contains supplementary material available at 10.1186/s13046-021-01856-3.

## Background

Triple-negative breast cancer (TNBC), characterized by lack of the estrogen receptor (ER), progesterone receptor (PR) and human epidermal growth factor receptor 2 (HER2) amplification, represents the most aggressive subtype of breast cancers [1–3]. Unlike patients with tumors that express hormone receptors or HER2, who have endocrine therapy and targeted therapy options, those with TNBC have limited treatment options [4–6]. In general, TNBC shows highly malignant features such as rapid recurrence, early onset age, greatly metastatic potential, and the most noticeable one is high proliferative potential [7–9]. This prominent biological characteristic means that a majority of patients with TNBC suffer rapid recurrence within 1 or 2 years after surgery or chemotherapy, which results in treatment failure and a short 5-year overall survival [2, 5, 10, 11]. However, the underlying mechanisms that drive the aggression of TNBC are not fully understood and it is imperative to explore new therapeutic targets against TNBC.

Increasing researches have suggested that the proto-oncogene MYC might have a vital function in aggressive breast cancers [12–14]. MYC serves as a transcription factor by forming a heterodimer with MYC associated factor X (MAX) and recruiting multiple co-activators and protein complexes to the E boxes of target genes, which is involved in multiple cellular processes, especially cell proliferation [15]. MYC promotes tumor progression by activating cell cycle related genes and repressing cyclin-dependent kinase (CDK) inhibitors, which contributes to uncontrolled proliferation and ultimately, the development of cancers [16, 17]. Furthermore, upregulation of MYC exists preferentially in TNBCs of the basal-like subtype, and elevated MYC signaling is associated with poor outcome [18, 19]. However, the mechanisms by which MYC is upregulated in TNBC, and how MYC promotes the malignant progress of TNBC remain unclear.

ACTL6A (Actin-like protein 6A), also known as BAF53a (53 kDa BRG-1/human BRM-associated factor), is involved in diverse cellular processes, such as vesicular transport, spindle orientation, nuclear migration, cell cycle and chromatin remodeling [20–22]. ACTL6A is also a component of the NuA4 histone acetyltransferase (HAT) complex that promotes transcriptional activation of selected genes principally by acetylation of nucleosomal histones H4 and H2A. Recently, ACTL6A has been reported to be related to the carcinogenesis of several tumors. It has been shown that ACTL6A interacts with tumor protein p63 (TP63) to repress WW and C2 domain containing 1 (WWC1), which is the regulator of the Hippo/yes associated protein (YAP) pathway, and ultimately contributes to regenerative proliferation and poor prognosis of head and neck squamous cell carcinoma [23]. Furthermore, ACTL6A plays an important role in maintaining the progenitor state in mouse epidermis as well as human keratinocyte by suppressing SWI/SNF-enabled activation of Kruppel like factor 4 (KLF4) and other epidermal differentiation genes [24]. In addition, Jeonghyeon Park et al. have reported that ACTL6A could form a nuclear cofactor complex with TIP49 and TIP48 and a distinct histone acetyltransferase (HAT) complex containing TRRAP, which is recruited by MYC to chromosomal sites. ACTL6A deletion mutants could inhibit the formation of these complexes to block the transformation of rat embryo fibroblasts by c-myc and H-ras and eliminate the HAT activity [25]. Although ACTL6A was characterized as an oncogenic driver in many human cancers, the underlying mechanisms remain limited.

In the present study, ACTL6A was first identified to be a stabilizer to MYC through blocking the interaction of MYC and GSK3β, and promoted the enrichment of MYC and KAT5 on the CDK2 promoters, increasing its transcriptional activity. In vivo experiments showed that co-therapy with CDK2 inhibitor and paclitaxel acted synergistically in tumor suppression of TNBC. These results uncovered a crucial role of ACTL6A in maintaining high-MYC level and suggested that targeting ACTL6A/MYC/CDK2 signaling might provide a novel and effective strategy to treat TNBC.

## Methods

### Cell culture

The 293FT cell line is a fast-growing, highly transfectable clonal isolate derived from human embryonal kidney cells transformed with the SV40 large T antigen, which were cultured in DMEM with 10% fetal bovine serum (FBS). Human breast cancer cell lines MCF-7, MDA-MB-468 and MDA-MB-231 were cultured in DMEM with 10% FBS; ZR-75-1, BT-474, and BT-549 cells were cultured in RPMI 1640 with 10% FBS; SKBR-3 were cultured in McCoy’s 5A medium with 10% FBS. The above cell lines were purchased from American Type Culture Collection (ATCC, Manassas, VA, USA). SUM159PT was purchased from Asterand Bioscience (Royston, UK) and cultured in Ham’s F-12 with 5% FBS, 1 μg/ml hydrocortisone, 5 μg/ml insulin and 10 mM HEPES. Cells were maintained at 37 °C in a 5% CO2 incubator. Cells were routinely tested for mycoplasma contamination using the Lookout Mycoplasma PCR Detection Kit (#MP0035; Sigma-Aldrich). Cells were treated with the proteasomal inhibitor MG132 (ApexBio, Hsinchu, Taiwan, China) at 10 μM for 5 h to inhibit proteasomal-mediated degradation and cycloheximide (CHX, ApexBio) at 50 μg/mL to inhibit translation. All cell lines were authenticated using short tandem repeat (STR) profiling before experiments.

### Immunoprecipitation (IP) assay

Lysates were prepared from the indicated cancer cells using a lysis buffer (150 mM NaCl, 10 mM HEPES, [pH 7.4], 1% NP-40), and the lysates were incubated with protein G agarose (IP04, Millipore), anti- ACTL6A antibody (#76682, CST), anti-MYC antibody (#9402, CST), Flag affinity agarose (A2220, Millipore) or HA affinity agarose (A2095, Millipore), overnight at 4 °C. Beads containing affinity-bound proteins were washed six times with the immunoprecipitation wash buffer (150 mM NaCl, 10 mM HEPES, [pH 7.4], and 0.1% NP-40), followed by elution with 1 M glycine [pH 3.0]. The eluates were then mixed with the sample buffer, denatured, and electrophoresed for western blotting analysis.

### Plasmids and generation of stably transfected cell lines

Human ACTL6A cDNA was PCR-amplified and cloned into the pLVX-IRES-Hyg vector (Clontech). Two shRNA against ACTL6A and one shRNA against MYC in pSuper-retro-neo vector were purchased from Sigma-Aldrich. Transfection of plasmids was performed using Lipofectamine 3000 reagent (Invitrogen, Carlsbad, California, USA) according to the manufacturer’s instructions. Cells (2 × 10^5^) were seeded and infected by retrovirus generated by pLVX-IRES-Hyg-ACTL6A and pSuper-retro-neo-ACTL6A-shRNA/-MYC-shRNA for 3 days. The stable cell lines expressing ACTL6A, ACTL6A-shRNAs and MYC-shRNA were selected with 200 μg/mL hygromycin and 400 μg/mL G418 for 7 days, respectively. The indicated sequences were provided in the Supplementary Table S1.

### Gene set enrichment analysis (GSEA)

Gene Set Enrichment Analysis (GSEA) is a computational method that determines whether an a priori defined set of genes shows statistically significant, concordant differences between two biological states (e.g. phenotypes). The results presented in the current manuscript use the two phenotypes divided by the median of ACTL6A mRNA expression of TCGA data. GSEA was performed using GSEA 2.0.9 (http://www.broadinstitute.org/gsea/) [26].

### The list of ACTL6A related genes from Cbioportal (**http://www.cbioportal.org/**)

The list of ACTL6A related genes comes from a publicly accessible online tool cbioportal and the data are from TCGA (http://www.cbioportal.org/). These genes were correlated with ACTL6A in mRNA levels. We selected genes with a positive correlation coefficient higher than 0.50 to form the ACTL6A-related gene set.

### Chromatin immunoprecipitation (ChIP)

Cells (2 × 10^6^) in a 100 mm culture dish were treated with 1% formaldehyde to cross-link proteins to DNA. The cell lysates were sonicated to the shear DNA to 300–1000 bp fragments. Equal aliquots of chromatin supernatants were incubated with 1 μg of an anti-MYC (#9402, CST), anti-KAT5 (ab23886, Abcam), anti-acetyl-histone H3 (Lys14) (D4B9) Rabbit mAb (#7627, CST), Anti-histone H4 (acetyl K5) (ab51997, Abcam), anti-histone H4 (acetyl K8) (ab15823, Abcam), anti-acetyl-histone H4 (Lys12) (D2W6O) Rabbit mAb (CST#13944), anti-acetyl-histone H4 (Lys16) (E2B8W) Rabbit mAb (CST#13534), and anti-IgG antibodies (Millipore, Billerica, MA, USA) overnight at 4 °C with rotation. After reversing the cross-linking of protein-DNA complexes to liberate the DNA, the human CDK2 promoter was amplified using real-time PCR. The primer sequences used are listed in Supplementary Table S1.

### Patient information and tissue specimens

We collected 344 cancer specimens that were histopathologically diagnosed at the Sun Yat-sen University Cancer Center from 2005 to 2013. All patients eligible for this study accepted surgery and were followed-up regularly. Clinical information of the samples is described in detail in Supplementary Table S2. The study was approved by the ethics committee of Sun Yat-sen University Cancer Center in accordance with the Declaration of Helsinki.

### Quantitative real-time PCR (qPCR)

Total RNA was isolated from cells or human tissue using the TRIzol reagent (Invitrogen, Carlsbad, CA, USA) according to manufacturer’s instructions. cDNA was synthesized from total RNA (2 μg) after adding RNase-free DNase. qPCR was performed in triplicate using 1 μL of cDNA in a standard SYBR premix Ex Taq (Takara, Shiga, Japan) on the CFX96 Real-Time PCR Detection System (Bio-Rad, Hercules, CA, USA). Glyceraldehyde-3-phosphate dehydrogenase (GAPDH) served as an internal control. The primer sequences used are listed in Supplementary Table S1.

### Western blotting

Total proteins of cells were extracted using Radioimmunoprecipitation assay (RIPA) lysis buffer (Thermo Scientific, Waltham, MA, USA) and were quantified by using a Bio-Rad DC protein assay kit II (Bio-Rad), separated by electrophoresis on 8–15% SDS-PAGE gels and electro transferred onto a Hybond ECL transfer membrane (Amersham Pharmacia, Piscataway, NJ, USA). The membranes were blocked with 5% skimmed milk and incubated with the appropriate antibodies. The antigen-antibody complexes on the membrane were detected using labeled secondary antibodies and enhanced chemiluminescence reagents (Thermo Scientific). The antibodies and their dilutions were as follows: anti-ACTL6A (sc-137,062, dilution 1:1000, Santa Cruz, CA USA), anti-MYC (#13987, dilution 1:1000, CST), anti-MYC (phospho T58/S62) (ab185655/ab185656, dilution 1:1000, Abcam, Cambridge, UK), CDK2 (#2546, dilution 1:1000, CST), HA (H6908, dilution 1:1000, Sigma-Aldrich), Flag (F7425, dilution 1:1000, Sigma-Aldrich), GAPDH (#5174, dilution 1:1000, CST), GSK3β (#9323, dilution 1:1000, CST), and then exposed to horseradish peroxidase (HRP)-conjugated secondary anti-mouse or rabbit antibodies. Protein expression was measured by using enhanced chemiluminescence (ECL) system (Amersham Pharmacia).

### Immunohistochemistry (IHC)

IHC was performed to detect the expression of ACTL6A in 344 breast cancer samples using the anti-ACTL6A antibody (sc-137,062, dilution 1:400, Santa Cruz). Two independent investigators who were blinded to the histopathologic features and clinical characteristics of the samples viewed and scored the immunostaining separately. The staining index (SI) of tissues was evaluated using the intensity and proportion of positively stained tumor cells. Staining intensity was classified as follows: 0, no staining; 1, weak staining (light yellow); 2, moderate staining (yellow brown); 3, strong staining (brown). Scores of positively stained cell proportions were: 0, no positive; 1, < 10%; 2, 10–35%; 3, 35–75%; 4, > 75%. Expression levels of the indicated proteins were determined using the staining index (SI). The SI consisted of possible scores of 0, 1, 2, 3, 4, 6, 8, 9 and 12. High and low expression of ACTL6A were then defined as SI ≥ 6 and SI < 6 respectively, according to the heterogeneity with the log-rank test statistics with respect to 5-year relapse-free survival.

### Xenograft tumor models

Female BALB/c-nu mice (5–6 weeks old, 18–20 g) were purchased and housed in barrier facilities on a 12-h light/dark cycle. The Institutional Animal Care and Use Committee of Sun Yat-sen University approved all the experimental procedures. To establish the subcutaneous xenograft model, 1 × 10^6^ SUM159PT-vector/scramble, −ACTL6A, −shACTL6A and -ACTL6A-shMYC cells were injected subcutaneously into the fat pads of 6 mice per group. One week after inoculation, the mice were treated with an intraperitoneal injection of K03861 (5 mg/kg, once a day for 5 consecutive days each week, for up to 7 weeks), PTX (10 mg/kg, once a day for 5 consecutive days each week, for up to 7 weeks), or both drugs for several weeks. The tumor volumes were determined every week. The tumor volume was calculated using the following equation: (L*W^2^) / 2 (L = length, W = width). The mice were sacrificed later, and the tumors were isolated and weighed. Serial 6.0-μm sections were cut and stained with anti-ACTL6A and anti-Ki-67 antibodies to determine the level of proliferation.

### Kaplan-Meier plotter database analysis

The prognostic significance of the mRNA expression of ACTL6A gene in breast cancer was evaluated using the Kaplan-Meier plotter (www.kmplot.com), an online database including gene expression data and clinical data [27]. With the purpose to assess prognostic value of ACTL6A in TNBC subgroup (the selected parameters were as follows: ER status (ER negative), PR status (PR negative), HER2 status (HER2 negative), and the analysis will run on 255 patients) and basal-like subtype (the selected parameter was Intrinsic subtype (basal), the analysis will run on 618 patients), the patient samples were divided into two cohorts according to the auto select best cutoff (TNBC subgroup: high-ACTL6A was 67 cases and low-ACTL6A was 188 cases; basal-like subtype: high-ACTL6A was 354 cases and low-ACTL6A was 264 cases). We analyzed the relapse-free survival (the selected parameter was Survival (RFS)) of TNBC and basal-like breast cancer patients by using a Kaplan-Meier survival plot. Briefly, the ACTL6A gene was uploaded into the database to obtain the Kaplan-Meier survival plots, in which the number-at-risk was shown below the main plot. Log rank *P*-value and hazard ratio (HR) with 95% confidence intervals were calculated and displayed on the webpage.

### Statistical analysis

Statistical analysis was performed using SPSS version 24.0 (SPSS Inc., Chicago, IL, USA). All data were described as means and standard deviations (SD). Statistical tests for data analysis included two-tailed Student’s t test, Mann-Whitney test and χ2 test. Kaplan-Meier methodology was used to evaluate survival probabilities and log-rank test was used to compare survival difference on univariate analysis. Multivariate statistical analysis was performed using a Cox regression model. *P* < 0.05 was considered to indicate statistical significance.

## Results

### ACTL6A interacts with and stabilizes MYC via inhibiting GSK3β-induced MYC degradation

**Fig. 1:**
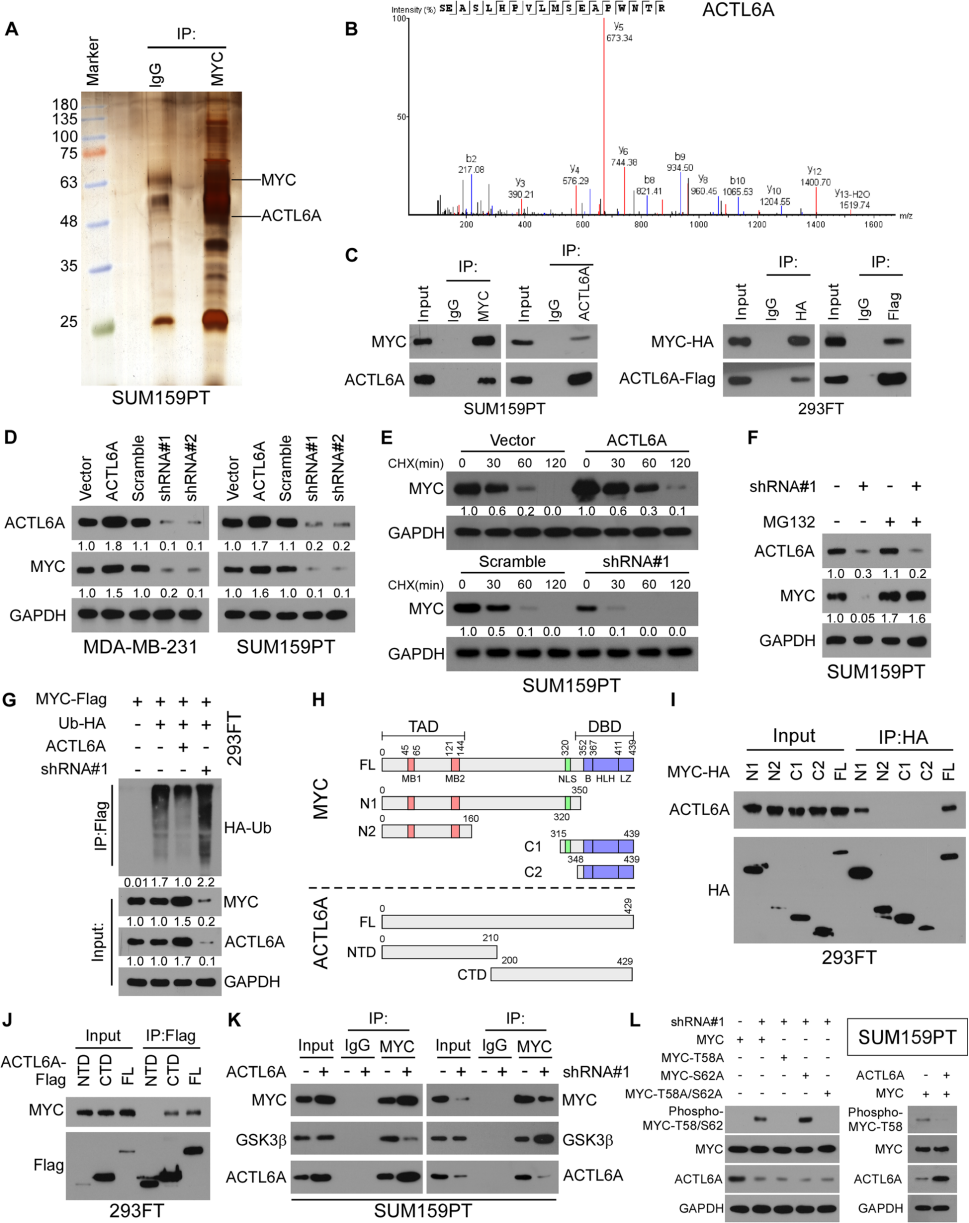
ACTL6A interacts with and stabilizes MYC protein via abrogating GSK3β-induced MYC degradation. **a** Lysates from SUM159PT cells were immunoprecipitated using an anti-MYC antibody, followed by MS peptide sequencing. ACTL6A was identified in the precipitate. **b** Representative MS plots and sequences of peptides from ACTL6A. **c** Reciprocal immunoprecipitation (IP) assay revealed the interaction between MYC and ACTL6A in SUM159PT and 293FT cells. **d** Western blot analysis of ACTL6A and MYC protein in control, ACTL6A-overexpressed and -knockdown SUM159PT cells. GAPDH was used as loading control. **e** Western blot analysis of MYC protein in the indicated cells treated with CHX (50 μg/mL) for 0, 30, 60, or 120 min. GAPDH was used as loading control. **f** MYC protein level in the indicated cells under MG132 (10 μM) treatment for 8 h and then western blotting was conducted. GAPDH was used as loading control. **g** Effect of ubiquitination on MYC by immunoprecipitation assay in SUM159PT cells. HA-tagged ubiquitin, ACTL6A-overexpressed plasmids, and ACTL6A shRNA plasmids were co-transfected into SUM159PT cells with Flag-tagged MYC plasmid. Immunoprecipitated proteins with anti-HA antibody were detected by MYC antibody and whole cell lysates were immunoblotted as loading controls. **h** The truncations of MYC and ACTL6A were based on known functional domain. **i, j** Detailed interactions between MYC and ACTL6A were analyzed by IP assays. **k** IP assay showed that overexpressing ACTL6A substantially decreased the interaction between MYC and GSK3β. **l** Transfected with wild-type MYC and different mutant MYC into SUM159PT cells and were analyzed by western blotting to detect the phosphorylated levels of MYC

We detected the MYC half-life in TNBC and non-TNBC cell lines and found that the half-life was prolonged in four TNBC cell lines (BT-549, MDA-MB-231, MDA-MB-468 and SUM159PT), compared with that in non-TNBC cells (MCF-7, BT-474, ZR-75-1 and SKBR-3), suggesting that the protein stability of MYC was upregulated in TNBC (Supplementary Figure 1A). To explore the mechanism by which a high level of MYC is retained, mass spectrometry (MS) was used to identify MYC-interacting proteins in a TNBC cell, SUM159PT. As shown in Fig. 1a and b, ACTL6A were identified as a potent MYC-interacting protein. Co-immunoprecipitation (Co-IP) using an anti-MYC antibody revealed that MYC bound to ACTL6A in SUM159PT cells. Reciprocally, IP assays using antibodies against ACTL6A further demonstrated that two proteins could form complex endogenously (Fig. 1c). Furthermore, Co-IP was performed in 293FT cell transfected with MYC-HA and ACTL6A-Flag, and confirmed that ACTL6A also interacted with MYC protein exogenously (Fig. 1c). Gene set enrichment analysis (GSEA) from The Cancer Genome Atlas (TCGA) database showed that high ACTL6A expression was significantly correlated with the MYC signature (Supplementary Figure 1B). These results suggested that ACTL6A might be involved in the regulation of MYC signaling.

We then chose MDA-MB-231 and SUM159PT in which MYC half-life was prolonged moderately, to establish ACTL6A-overexpressed or -silenced cell lines. We detected ACTL6A and MYC expression in the above stable transfected cell lines and found that overexpression of ACTL6A elevated, while knockdown of ACTL6A decreased MYC protein levels; however, the altered-ACTL6A expression did not affect the mRNA levels of MYC (Fig. 1d and Supplementary Figure 1C). To figure out whether ACTL6A regulates the stability of MYC protein, we detected its protein levels in the presence of cycloheximide (CHX) and MG132, respectively. It’s interesting that the MYC half-life was remarkably prolonged under these treatments in ACTL6A-upregulated TNBC cells, compared with that in the vector group, and the opposite effects were observed in the ACTL6A-silenced cells (Fig. 1e and f, Supplementary Figure 1D-G). In addition, we found that high levels of ACTL6A inhibited ubiquitination of MYC and low ACTL6A levels promoted this process (Fig. 1g and Supplementary Figure 1H). Furthermore, subcellular IP assays indicated that ACTL6A could bind to MYC in the nucleus and cytoplasm (Supplementary Figure 1I). The subcellular fraction assay was performed in TNBC cells with or without ACTL6A overexpression and showed that upregulation of ACTL6A promoted evident accumulation of MYC in the nucleus and cytoplasm, suggesting that ACTL6A stabilized MYC independent of localization (Supplementary Figure 1 J). Therefore, these results confirmed that ACTL6A bound to and stabilized MYC by decreasing its ubiquitination.

To explore the specific binding domain between ACTL6A and MYC, truncations of the two genes were constructed (Fig. 1h). IP assays showed that only those MYC truncations with the N1 domain could bound to ACTL6A, suggesting that the interaction between ACTL6A and MYC required the N-terminal domains (Fig. 1i). In addition, the C-terminal domain (CTD) of ACTL6A was necessary for the interactions with MYC (Fig. 1j). The degradation of MYC is largely dependent on the phosphorylation at threonine 58 (phospho T58), which is mediated by GSK3β-interaction [28–31]. Furthermore, we found that overexpression of ACTL6A substantially inhibited the interaction between MYC and GSK3β, while downregulating ACTL6A strengthened the MYC-GSK3β interaction (Fig. 1k). To detect the phosphorylation levels of MYC under low-ACTL6A expression, we transfected SUM159PT cells with wild-type MYC and MYC mutants (MYC-T58A, MYC-S62A and MYC-T58A/S62A), respectively. The results showed that depletion of ACTL6A increased the phosphorylation level of wild-type MYC and MYC-S62A, whereas phosphorylated MYC-T58A and MYC-T58A/S62A were not detected (Fig. 1l). In addition, we detected MYC phosphorylation using Anti-c-Myc (phospho T58), and found that ACTL6A overexpression suppressed MYC T58 phosphorylation, compared to control group (Fig. 1l). Moreover, we established the SUM159PT-MYC-T58A-Flag cell lines and detected the half life of MYC followed altered ACTL6A expression, and the results showed that stability of MYC-T58A was not affected by ACTL6A (Supplementary Figure 1 K). Taking these results together, we found that ACTL6A enhanced MYC stability via binding to its N1 domain, impairing the MYC-GSK3β interaction and abrogating MYC phosphorylation on T58.

### ACTL6A enhances the transcriptional activity of CDK2 by increasing the enrichment of MYC and KAT5 on its promoters

**Fig. 2:**
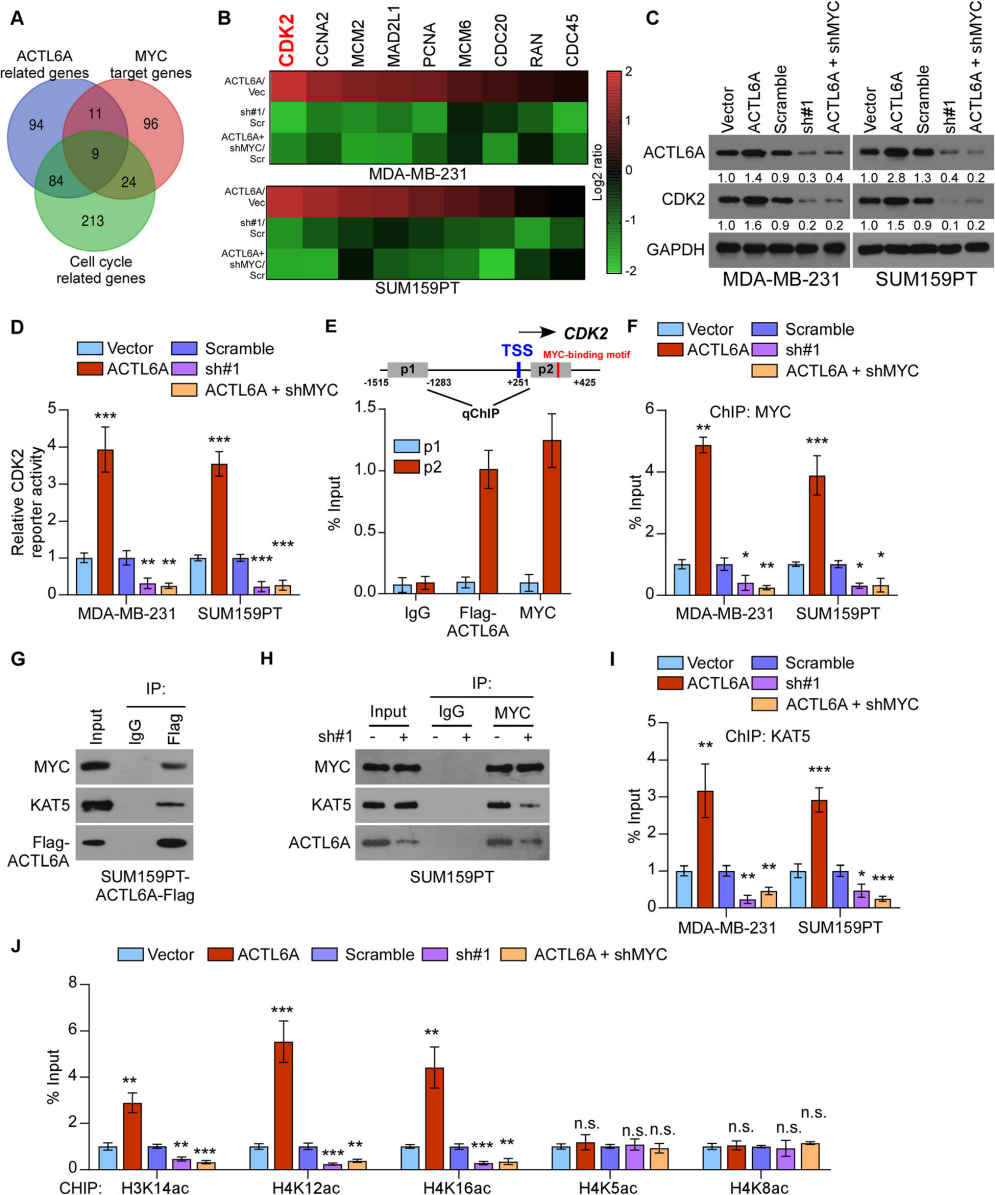
ACTL6A enhances CDK2 transcriptional activity by increasing the enrichment of MYC and KAT5 on its promoters. **a** A Venn diagram among MYC related genes, ACTL6A related genes, and cell cycle related genes. **b** Real-time PCR analysis of the expression of 9 genes in SUM159PT cells with altered ACTL6A expression. **c** Western blot analysis of CDK2 in indicated cells. **d** Luciferase activity of CDK2 reporter were detected in the indicated cells. **e** Schematic illustration of the PCR fragments of the human CDK2 gene promoters (upper panel). Chromatin immunoprecipitation (ChIP) assays were performed in SUM159PT-ACTL6A-Flag cells using antibodies against Flag and MYC to identify the occupancy on CDK2 gene promoters. Immunoglobulin G (IgG) was used as a negative control. **f** Enrichment of MYC on p2 fragment of CDK2 promoter. **g** IP assay revealed that ACTL6A formed complex with MYC and KAT5 in SUM159PT-ACTL6A cells. **h** Silencing of ACTL6A abrogated interaction between MYC and KAT5. **(I)** Enrichment of KAT5 on p2 fragment of CDK2 promoter. **j** Appearance of acetylated histone H3K14ac and histone H4 (K12ac, K16ac, K5ac and K8ac) on p2 fragment of CDK2 promote in the indicated cells. Each bar represents the mean ± S.D. of three independent experiments. Two-tailed Student’s t test was used. *P < 0.05, **P < 0.01, ***P < 0.001 and n.s. for no significance

To further explore the mechanism by which ACTL6A regulates MYC signaling, we drew a Venn diagram using the lists of ACTL6A-related genes from cBioportal (http://www.cbioportal.org/), hallmarks of MYC-related genes and cell cycle related genes from GSEA, respectively. Interestingly, 9 genes were commonly involved in the three gene sets (Fig. 2a). We then detected the expression of these genes and found that CDK2, a typical downstream target gene of MYC, was significantly increased along with high-ACTL6A expression, and reduced in low-ACTL6A level among the detected genes (Fig. 2b). Upregulated ACTL6A increased the protein levels of CDK2, while knockdown of ACTL6A exhibited the reverse effects (Fig. 2c and Supplementary Figure 2A). Luciferase reporter assays revealed that ACTL6A overexpression activated the luciferase activity of the CDK2 promoters in TNBC cells, whereas ACTL6A downregulation attenuated it (Fig. 2d). Furthermore, the ChIP assay revealed that ACTL6A-Flag and MYC both interacted with the p2 fragment, which contains a MYC-specific DNA-binding motif (CACGTG) [32–34], not the p1 fragment, which is 1500 bp upstream away from the CDK2 promoters, indicating that ACTL6A could bind on the target gene promoters (Fig. 2e). Furthermore, we performed ChIP assay using the p2 fragment and found that ACTL6A overexpression increased, but silencing of ACTL6A significantly reduced the occupancy of MYC on the CDK2 promoters, indicating that ACTL6A played an important role in the transcription of CDK2 (Fig. 2f).

Previous studies have showed that KAT5 possess histone acetyltransferase (HAT) activity and plays significant roles in transcription [35, 36]. Moreover, studies have reported that MYC recruits KAT5 onto the promoters of target genes in the transcription process. IP assays showed that ACTL6A could form a complex with KAT5 and MYC, while knockdown of ACTL6A impaired the interaction between MYC and KAT5 (Fig. 2g and h). The ChIP assay using the p2 fragment confirmed that upregulating ACTL6A promoted, while silencing of ACTL6A inhibited the recruitment of KAT5 on the CDK2 promoters (Fig. 2i). Furthermore, we performed ChIP using the p2 fragment and found that upregulating ACTL6A enhanced, while knockdown of ACTL6A markedly reduced the appearance of acetylated histone H3 (K14) and H4 (K12, and K16) at the proximal promoter of the CDK2 gene, which has been reported to be acetylated by KAT5 to increase the DNA accessibility [37] (Fig. 2j), but not H4 (K5 and K8). In addition, silencing MYC in ACTL6A-overexpressed cell lines blocked the transcriptional activity of CDK2, suggesting that MYC was essential for the ACTL6A-induced transcriptional regulation (Fig. 2b-d, f, i and j). Therefore, these results indicated that ACTL6A contributed to the enrichment of MYC and KAT5 on the CDK2 promoters and enhanced CDK2 transcription by facilitating histone acetylation.

### ACTL6A promotes cell proliferation and tumorigenicity via MYC signaling in TNBC cells in vitro and in vivo

**Fig. 3:**
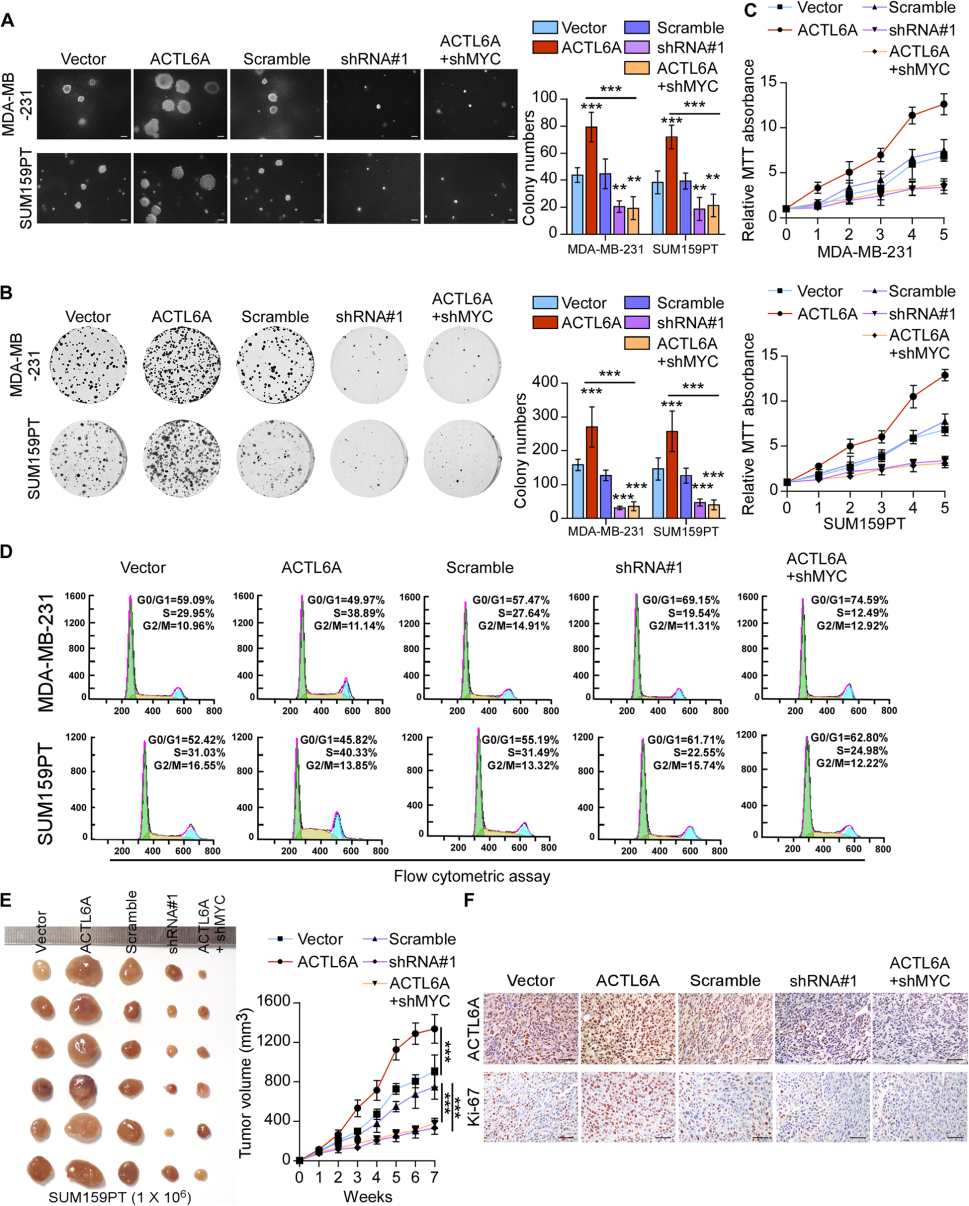
ACTL6A promotes cell proliferation and tumorigenicity via MYC signaling in TNBC cells. **a** Representative images (left) and quantification (right) of anchorage-independent growth colony formation for the indicated cell lines. Scale bars represent 200 μm. **b** Representative images (left) and quantification (right) of colony formation for the indicated cell lines. **c** MTT assays showed the cell proliferative capacity. **d** Flow cytometric analysis of TNBC cells with altered ACTL6A expression. **e** Vector/scramble, ACTL6A-overexpressed, ACTL6A-silenced or ACTL6A-shMYC SUM159PT cell lines were subcutaneously injected into mice (1 × 10^6^/injection, *n* = 6/group). The tumor volumes in each group are shown. **f** IHC of Ki-67 staining showed in the indicated xenografts. Scale bars represent 50 μm. Data represent the means ± S.D. of three independent experiments. Two-tailed Student’s t test was used. **P < 0.01, ***P < 0.001

To further investigate the biological role of ACTL6A in the proliferative activity of TNBC, we performed soft agar, colony formation and MTT assays. As shown in Fig. 3a-c, overexpression of ACTL6A significantly promoted, while knockdown of ACTL6A suppressed cell proliferation. Moreover, we examined the effect of ACTL6A on cell-cycle progression using flow cytometry. Cell lines with up-regulated ACTL6A expression increased proportions of cells in the S phase and reduced proportions of cells in the G1 phase significantly, while silencing ACTL6A had the opposite effects (Fig. 3d and Supplementary Figure 3A). We found that silencing MYC could reverse the ACTL6A-induced proliferative effects in TNBC cell lines, determining that MYC signaling was essential for the proliferative promotion in high-ACTL6A TNBC cells (Fig. 3a-d).

Next, we assessed the effects of ACTL6A on tumorigenicity in vivo. We generated xenografts through subcutaneous injection of SUM159PT stable cell lines and the tumor growth was measured. The ACTL6A-overexpressing group had obviously enhanced, whereas knockdown of ACTL6A suppressed tumor growth, both as compared with the vector group (Fig. 3e and Supplementary Figure 3B). Moreover, tumors from the high-ACTL6A group exhibited high expression of the proliferation marker Ki-67 compared with that in the vector group. By contrast, downregulation of ACTL6A was associated with low Ki-67 expression (Fig. 3f and Supplementary Figure 3C). As expected, blockage of MYC signaling suppressed the ACTL6A-promoting effects on tumorigenicity of TNBC cells (Fig. 3e and f, Supplementary Figure 3B and C). Furthermore, we tranfected 0.2 μg plasmids of wild-type MYC and MYC-T58A in MDA-MB-231-shACTL6A and SUM159PT-shACTL6A cells, respectively, to make the mRNA level of MYC equal, but the protein levels were different. Ectopic expression of MYC-T58A rescued the inhibition of anchorage-independent growth colony formation and colony formation caused by silencing ACTL6A while wild-type MYC failed to rescue (Supplementary Figure 3D). These results indicated that ACTL6A enhanced the highly proliferative activity of TNBC cells via MYC signaling.

### Combination of K03861 (CDK2 inhibitor) and paclitaxel results in synergistic growth inhibition of TNBC cells with high-ACTL6A level in vitro and in vivo

**Fig. 4:**
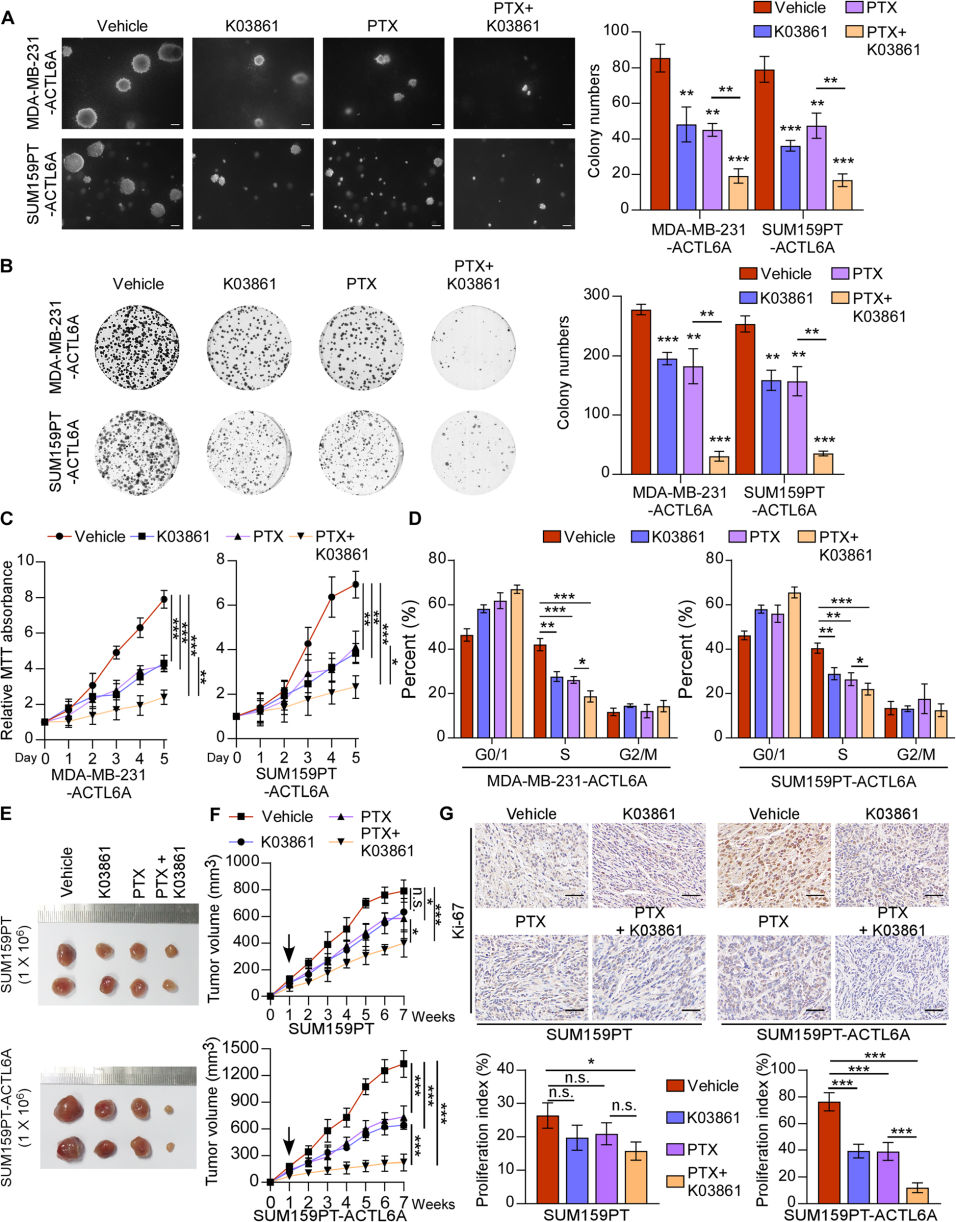
Combination of K03861 and paclitaxel results in synergistic growth inhibition in TNBC cells with high-ACTL6A. **a-c** Soft agar assay, colony formation assay and MTT assay were performed in vehicle, K03861 (50 nM), PTX (100 nM) or combination of K03861 and PTX groups in ACTL6A overexpressing cells**.** Scale bars represent 200 μm. **d** Flow cytometric analysis of the indicated cells with different treatments. **e** SUM159PT cells with or without ACTL6A overexpressing were subcutaneously injected into mice (1 × 10^6^/injection, *n* = 6/group). One week after inoculation, the mice were intraperitoneally injected with K03861 (5 mg/kg, once a day for 5 consecutive days each week, for up to 7 weeks), PTX (10 mg/kg, once a day for 5 consecutive days each week, for up to 7 weeks), combination with two drugs or vehicle. **f** The tumor volumes in each group are shown. **g** IHC of Ki-67 staining showed in the indicated xenografts. Scale bars represent 50 μm. Data represent the means ± S.D. of three independent experiments. Two-tailed Student’s t test was used. *P < 0.05, **P < 0.01, ***P < 0.001

Given the mechanism that ACTL6A activated the MYC/ CDK2 axis, we hypothesized that a CDK2 inhibitor would have potentially therapeutic effects against TNBC in combination with the first-line chemotherapy drug of breast cancer, paclitaxel (PTX). As shown in Fig. 4a-d, SUM159PT- and MDA-MB-231-ACTL6A cells treated with K03861 and PTX were decreased in proliferative activity markedly, while weak effects were observed in cells treated with K03861 or PTX alone, compared with vehicle group. In addition, a slightly repressive effect was shown in cell proliferation of MDA-MB-231 and SUM159PT cells, compared with ACTL6A-overexpressing cells under the above treatments. Afterwards, we injected SUM159PT cells with or without overexpressing-ACTL6A into mice subcutaneously and observed the tumor growth in each experimental group with different treatments. The results showed that the tumors treated with K03861 and PTX were much smaller than those in other group, whereas treatment with either K03861 or PTX alone showed a slight inhibition in tumor growth compared with the control group (Fig. 4e and f). IHC of Ki-67 in tumor specimens was performed, which showed that co-therapy with K03861 and PTX markedly reduced the Ki-67 staining intensity in ACTL6A overexpressing tumors among these groups (Fig. 4g). The expression of CDK2 and p-CDK2 (Thr160) were decreased under the treatment of K03861 in the ACTL6A overexpressing subcutaneous tumors (Supplementary Figure 4E). The treatment of K03861, PTX, or combined therapy, only slightly repressed tumorigenicity in SUM159PT cells, compared with that in ACTL6A-overexpressing SUM159PT cells (Fig. 4e and f, Supplementary Figure 4E). However, there was no significant efficacy in TNBC cells with knockdown of ACTL6A and in non-TNBC cells (Supplementary Figure 5A-F and Supplementary Figure 6A-F). Furthermore, we transfected 1 μg plasmids of wild-type MYC and MYC-T58A in MDA-MB-231-ACTL6A and SUM159PT-ACTL6A cells, respectively, which were treated with K03861, PTX, or combined therapy. The results showed that MYC-T58A could induce resistance to the above treatments, while wild-type MYC could not in anchorage-independent growth colony formation and colony formation assay (Supplementary Figure 6G and H), which further supported that TNBC patients with high-ACTL6A could benefit from therapy with CDK2 inhibition. Collectively, these results suggested that the combination treatment of CDK2 inhibitor and chemotherapeutic drug might have promoting effects in anti-TNBC with high-ACTL6A level.

### ACTL6A/MYC/CDK2 axis is upregulated in TNBC tissues and cell lines

**Fig. 5:**
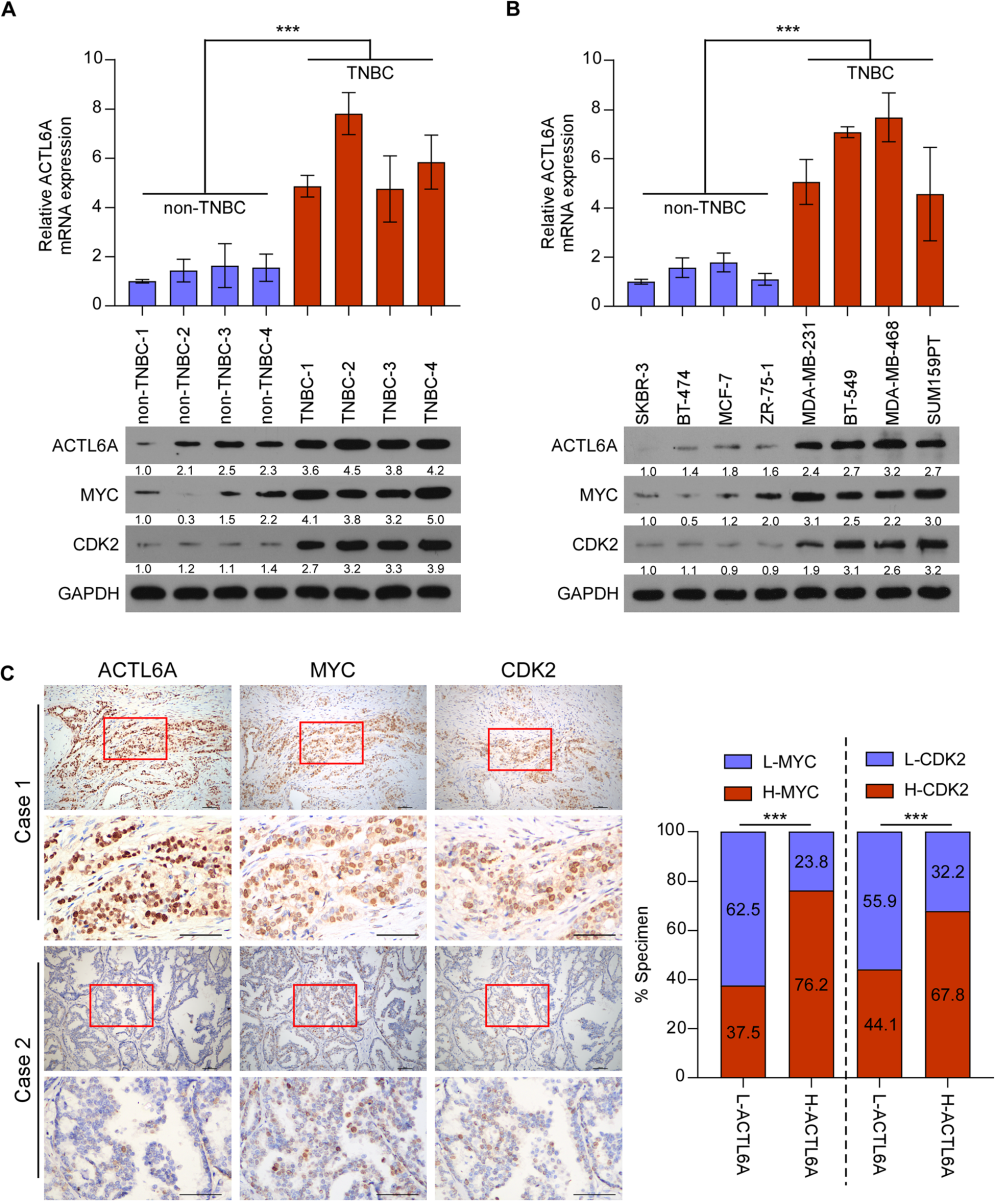
ACTL6A/MYC/CDK2 axis is upregulated in TNBC tissues and cell lines. **a** and **b)** Real-time PCR and western blotting analysis of ACTL6A, and western blotting analysis of MYC and CDK2 expression in human breast cancer tissues (**a**) and cell lines (**b**). GAPDH was used as a loading control. **c** Representative images of ACTL6A, MYC and CDK2 IHC staining in the same batch of breast cancer patient specimens. Scale bars represent 50 μm. Right panel, correlation analysis revealed that ACTL6A expression was significantly associated with MYC and CDK2 expression in patient specimens (χ^2^ test). ***P < 0.001

To explore the role of ACTL6A/MYC/CDK2 in the progression of TNBC, we detected these genes in breast cancer tissues and cell lines. Both mRNA and protein levels of ACTL6A were significantly upregulated in TNBC samples and cell lines compared with those in non-TNBCs, while MYC and CDK2 protein levels mirrored those of ACTL6A (Fig. 5a and b). Moreover, we analyzed the mRNA expression of ACTL6A and CDK2, the protein expression of MYC in public human breast cancer datasets from TCGA. The results revealed that the expression of the three genes were significantly upregulated in TNBC samples compared with non-TNBC tissues (Supplementary Figure 7A-C). IHC staining for the three genes was performed in the same batch of clinical samples and we found that ACTL6A expression was positively and significantly associated with MYC and CDK2 protein expression, suggesting that the ACTL6A/MYC/CDK2 axis in our study was clinically relevant (Fig. 5c). These results suggested a substantial increase of ACTL6A, MYC and CDK2 expression in TNBC.

### High expression of ACTL6A is associated with poor prognosis in TNBC patients

**Fig. 6:**
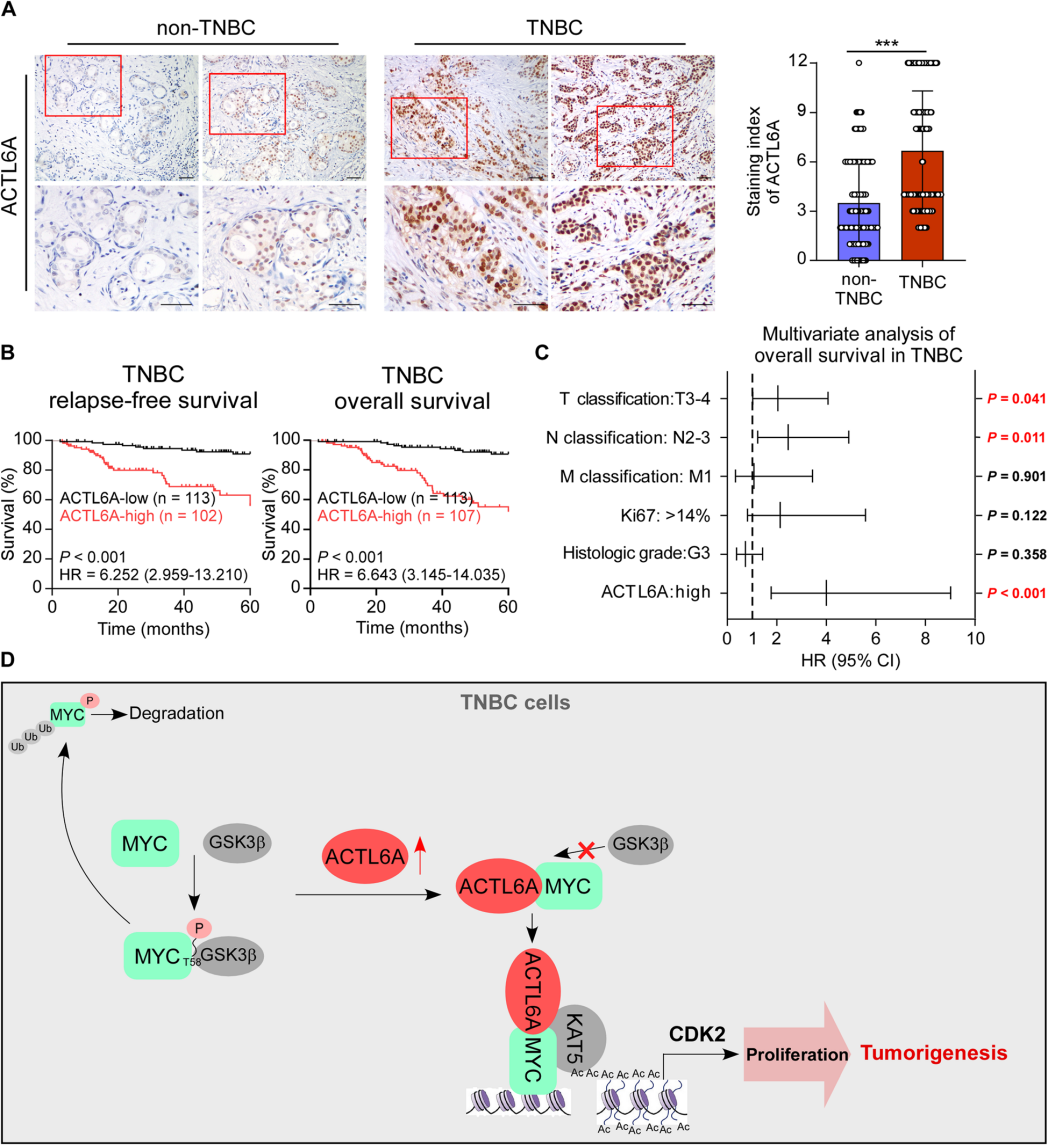
High expression of ACTL6A is associated with poor prognosis in TNBC patients. **a** Representative images of ACTL6A staining in non-TNBC and TNBC specimens. The staining index of ACTL6A in non-TNBC and TNBC group was indicated. Mann-Whitney test was used. Scale bars represent 50 μm. **b** Kaplan-Meier analysis of RFS and OS for TNBC patients with low expression of ACTL6A versus high expression of ACTL6A (*P* < 0.001). **c** Multivariate Cox regression analysis to evaluate the significance of the association between ACTL6A signature and OS in the presence of other clinical variables. **d** Proposal model. ***P < 0.001

The clinical significance of high ACTL6A expression was further assessed using immunohistochemistry (IHC) staining in 344 archived breast cancer tissue samples, including 124 non-TNBC cases and 220 TNBC cases (Supplementary Table S2). The staining index (SI) of ACTL6A was calculated based on both the staining intensity and the proportion of positive cells, and the SI of ACTL6A was significantly higher in TNBC specimens than in non-TNBC tissues (Fig. 6a). IHC analysis revealed that 29.8% showed high-expression and 70.2% showed low-expression of ACTL6A among the non-TNBC samples, while 48.6% showed high-expression and 51.4% showed low-expression of ACTL6A among the TNBC specimens (Supplementary Figure 8A). We then analyzed the correlation between ACTL6A expression and the clinicopathological characteristics of patients with breast cancer and found that high ACTL6A levels were associated with T, N classification and the Ki-67 index (Supplementary Table S3). Furthermore, univariate analysis indicated that TNBC patients with high-ACTL6A suffered shorter 5-year relapse-free survival (RFS, hazard ratio (HR) = 6.252, 95% confidence interval (CI) 2.959–13.210, *P* < 0.001) and overall survival (OS, HR = 6.643, 95% CI 3.145–14.035, *P* < 0.001) (Fig. 6b and Supplementary Table S4). In addition, we searched for survival information in the Kaplan-Meier Plotter database (http://kmplot.com/analysis) and found that patients with high expression of ACTL6A exhibited shorter RFS compared with those with low-ACTL6A in TNBC and basal-like subgroup (Supplementary Figure 8B). Multivariate Cox regression analysis indicated that high-ACTL6A expression and T, N classification were recognized as independent prognostic factors for the 5-year overall survival in TNBC subtype (Fig. 6c). However, ACTL6A expression could not predict poor RFS or OS in patients with non-TNBC (Supplementary Figure 8C). These results suggested that upregulation of ACTL6A might be involved in TNBC progression, leading to a poor clinical outcome.

## Discussion

The present study reveals that ACTL6A/MYC/CDK2 axis is specifically up-regulated in TNBC, which contributes to its high proliferative activity. Furthermore, we found that ACTL6A interacted with MYC to protect it from GSK3β-induced degradation and enhanced the recruitment of MYC and KAT5 on the CDK2 promoters, increasing its transcriptional activity (Fig. 6d). It was important to note that combination of CDK2 inhibitor (K03861) and paclitaxel, the first-line chemotherapeutic drug of TNBC, resulted in synergistic tumor growth inhibition of TNBC cells. Therefore, our results suggest that pharmacological targeting ACTL6A/MYC/CDK2 axis might be an efficient strategy against TNBC.

In clinical studies, patients with triple-negative tumors have been found to respond to neoadjuvant chemotherapy with equal or better efficacy than those with receptor positive tumors, presumably as a result of the higher proliferative activity [6, 38]. Because of this malignant characteristic, patients with TNBC have a tendency to experience chemoresistance and early relapse. However, the mechanism for the poor prognosis of this subtype remains unclear. In the present study, we found that MYC signaling was hyperactive in TNBC and contributed to its high proliferative activity, which was mediated by upregulated ACTL6A. ACTL6A increased the stability of MYC protein and promoted the recruitment of MYC on the promoters of CDK2, leading to hyperactivation of CDK2 transcription in TNBC cell lines. Importantly, combination of CDK2 inhibitor (K03861) and PTX, the first-line drug of TNBC, resulted in synergistic tumor growth inhibition of TNBC cells. Therefore, our results shed light on the mechanism of ACTL6A in regulation of MYC and suggest that blockage of MYC signaling might show a potential role in curing TNBC.

CDKs play important parts in cell cycle progression, which is a pivotal process for the uncontrolled growth of cancer cells and thus serve as a potential therapy target. However, several clinical trials have declared the failure of CDK inhibitors because of their limited clinical effect [39–41]. This may have been due to lack of target specificity or absence of patient selection based on clinically relevant biomarkers [42]. In our study, we found that TNBC cells with high-ACTL6A expression were more sensitive to CDK2 inhibitor, K03861, while those with low-ACTL6A expression and non-TNBC cells did not. Moreover, in vivo assays also confirmed these findings that K03861 took no effects on tumor growth in mice injected with SUM159PT-shACTL6A and non-TNBC cells. Therefore, it seems advisable that ACTL6A could serve as a marker to subgroup TNBC patients. Patients with high-ACTL6A expression might be suggested to take K03861 as an alternative therapy, while K03861 might not be a good choice for those with low-ACTL6A. Collectively, CDK inhibitors are reported not to be suitable for all patients, however, our results uncover the crucial role of ACTL6A in identifying a subset of breast cancer patients who would benefit from therapy with K03861. Meanwhile, more TNBC samples should be collected to further examine the correlation between ACTL6A expression level and the effectiveness of K03861.

Notably, it has been reported that MYC is preferentially overexpressed in high-grade breast cancer, especially in TNBC, and increased MYC activity is associated with poor outcome [43, 44]. TNBC exhibits a high-MYC expression profile due to mechanisms such as copy number amplification, changes in MYC promoter transcriptional regulation and protein stability. Because MYC is a short-lived protein, so the regulation of protein stability seems to be the most critical regulation in MYC oncogenic functions. However, the mechanism by which MYC retains its high expression in TNBC remains unclear. In our research, we identified ACTL6A was a potent MYC-interacting protein using mass spectrometry. The results showed that ACTL6A stabilized MYC via directly binding to its N terminus, which abrogated GSK3β-induced phosphorylation and degradation, demonstrating that the high-MYC level reflected prolonged protein stability. Collectively, these data reveal a novel mechanism of MYC overexpression which was mediated by ACTL6A upregulation in TNBC specimens.

Although it has been reported that ACTL6A functions as a component of nuclear cofactor complexes recruited by MYC and it could form a HAT complex with HATs, TRRAP and TIP60, and ACTL6A deletion mutants inhibits the formation of the complex to block the transformation of rat embryo fibroblasts by c-myc and H-ras [25, 45], our data explored completely new roles of ACTL6A in regulation of MYC functions. The results showed that ACTL6A enhanced MYC stability via binding to the CTD in ACTL6A, impairing the MYC-GSK3β interaction and abrogating MYC phosphorylation on T58. In addition, we found that ACTL6A-MYC complex preferentially recruited another HAT, KAT5, not TRRAP or TIP60, which potently increased CDK2 transcriptional activity. Different from the previous reports, the novel oncogenic functions of ACTL6A was investigated in TNBC cell lines, suggesting it may be distinct regulation in human cancers.

Recently, the carcinogenesis of ACTL6A has been reported in several tumors, including hepatocellular carcinoma, squamous cell carcinoma, colon cancer, glioma, osteosarcoma and cervical cancer [23, 46–50]. However, whether ACTL6A takes part in the tumorigenesis of breast cancer, especially TNBC, remains unclear. Herein, we demonstrated that ACTL6A maintained the high proliferative activity of TNBC via interacting with MYC to protect it from GSK3β-induced degradation and enhancing MYC to recruit on the CDK2 promoters, which activated its transcription. Both in vitro and in vivo assays showed that ACTL6A promoted the proliferation of TNBC. Furthermore, ACTL6A was upregulated in both TNBC tissues and cell lines, which correlated with poor prognosis. These results suggest that ACTL6A enhanced tumor growth via accelerating the cell cycle process and thereby contributing to the high proliferative activity and rapid recurrence of TNBC.

## Conclusions

The present study reveals that ACTL6A/MYC/CDK2 axis is specifically up-regulated in TNBC, which contributes to its high proliferative activity. Furthermore, we found that ACTL6A increased the stability of MYC protein and promoted the recruitment of MYC on the promoters of CDK2, leading to hyperactivation of CDK2 transcription in TNBC cell lines. In vivo experiments demonstrated that co-therapy with blocking agent of MYC signaling and chemotherapeutic drugs showed synergy in tumor suppression of TNBC. These data uncover a crucial role of ACTL6A in maintaining high-MYC level and propose that targeting ACTL6A/MYC/CDK2 signaling might provide a novel and effective strategy to cure TNBC.

## Supplementary Information

**Additional file 1: Table S1.** Primers. **Table S2**. Clinicopathological characteristics of 344 breast cancer patients and 220 triple-negative breast cancer patients. **Table S3**. Correlation between ACTL6A and clinicopathological characteristics of breast cancer patients. **Table S4**. Univariate and multivariate analysis of factors associated with 5-year overall survival and relapse-free survival in patients with TNBC.

**Additional file 2: Figure S1. (A)** Western blotting analyzed the MYC expression in 4 TNBC and 4 non-TNBC cell lines treated with CHX (50 μg/mL) for 0, 30, 60, or 120 min. GAPDH was used as loading control. The right panel was the statistical analysis. The MYC half-life’s was about 37 min in BT-549, 34 min in MDA-MB-231, 45 min in MDA-MB-468, 32 min in SUM159PT, 19 min in MCF-7, 19 min in BT-474, 20 min in ZR-75-1, and 20 min in SKBR-3. **(B)** Gene set enrichment analysis (GSEA) of TCGA dataset showed significant enrichment of MYC signature (HALLMARK_MYC_TARGETS_V1), in samples with high expression of ACTL6A. NES, normalized enrichment score. **(C)** Real-time PCR analysis of MYC in control, ACTL6A-overexpressed and -knockdown SUM159PT cells. **(D)** The statistical graph of Fig. 1e. The MYC half-life’s was about 45 min in ACTL6A-overexpressing group, 34 min in vector, 31 min in scramble groups, and 17 min in shACTL6A group. **(E)** The statistical graph of Fig. 1f. **(F)** Western blotting analyzed the MYC expression in MDA-MB-231 cells treated with CHX (50 μg/mL) for 0, 30, 60, or 120 min. GAPDH was used as loading control. The lower panel was the statistical analysis. The MYC half-life was about 50 min in ACTL6A-overexpressing group, 36 min in vector group, 31 min in scramble groups, and 14 min in shACTL6A group. **(G)** MYC protein level in the indicated cells under MG132 (10 μM) treatment for 8 h and then western blotting was conducted. GAPDH was used as loading control. **(H)** The statistical graph of Fig. 1g. **(I)** Subcellular IP assays were performed using anti-ACTL6A antibody in SUM159PT cells. C, cytoplasm; N, nucleus. **(J)** Immunoblot for the indicated proteins of subcellular fractions in SUM159PT cells with or without ACTL6A overexpression. Lamin A/C and GAPDH expression were used as nuclear and cytoplasmatic controls, respectively. **(K)** Western blotting analyzed the MYC expression in SUM159PT-MYC-T58A-Flag cell lines treated with CHX (50 μg/mL) for 0, 30, 60, or 120 min. GAPDH was used as loading control. The right panel was the statistical analysis. The MYC half-life was about 51 min in ACTL6A-overexpressing group, 55 min in vector group, 62 min scramble groups, and 62 min in shACTL6A group. Two-tailed Student’s t test was used. ***P < 0.001 and n.s. stands for no significance.

**Additional file 3: Figure S2. (A)** The statistical graph of Fig. 2c. ***P < 0.001

**Additional file 4: Figure S3. (A)** The percentage of the indicated cells in G0/G1, S and G2/M phases was analyzed in flow cytometric analysis. **(B)** The tumor weights in the indicated groups are measured. **(C)** Percentage of Ki-67 were shown in the indicated cells. **(D)** Quantification of anchorage-independent growth colony formation and colony formation for the indicated cell lines. Two-tailed Student’s t test was used. *P < 0.05, **P < 0.01 and ***P < 0.001.

**Additional file 5: Figure S4. (A-C)** Soft agar assay, colony formation assay and MTT assay were performed in vehicle, K03861 (50 nM), PTX (100 nM) or combination of K03861 and PTX groups in TNBC cells. **(D)** Flow cytometric analysis of the indicated cells with different treatments. **(E)** Immunoblot for the CDK2 and p-CDK2 (Thr160) proteins from SUM159PT and SUM159PT-ACTL6A tumors. GAPDH was used as loading control. *P < 0.05, **P < 0.01 and n.s. stands for no significance.

**Additional file 6: Figure S5. (A-C)** Soft agar assay, colony formation assay and MTT assay were performed in vehicle, K03861 (50 nM), PTX (100 nM) or combination of K03861 and PTX groups in silencing ACTL6A cells**. (D)** Flow cytometric analysis of the indicated cells with different treatments. **(E)** ACTL6A-silenced cell lines SUM159PT were subcutaneously injected into mice (1 × 10^6^/injection, *n* = 6/group). One week after inoculation, the mice were intraperitoneally injected with K03861 (5 mg/kg, once a day for 5 consecutive days each week, for up to 7 weeks), PTX (10 mg/kg, once a day for 5 consecutive days each week, for up to 7 weeks), combination with two drugs or vehicle. The tumor volumes in each group are shown. **(F)** IHC of Ki-67 staining showed in the indicated xenografts. Data represent the means ± S.D. of three independent experiments. Two-tailed Student’s t test was used. *P < 0.05, **P < 0.01 and n.s. stands for no significance.

**Additional file 7: Figure S6. (A-C)** Soft agar assay, colony formation assay and MTT assay were performed in ZR-75-1 and SKBR-3 cells treated with vehicle, K03861 (50 nM), PTX (100 nM) or combination with two drugs. **(D)** Flow cytometric analysis of the indicated cells with different treatments. **(E)** ZR-75-1 cells were subcutaneously injected into mice (1 × 10^6^/injection, n = 6/group). One week after inoculation, the mice were intraperitoneally injected with K0386, PTX, combination with two drugs or vehicle. The tumor volumes in each group are shown. **(F)** IHC of Ki-67 staining showed in the indicated xenografts. **(G-H)** Quantification of anchorage-independent growth colony formation (G) and colony formation (H) for the indicated cell lines. Data represent the means ± S.D. of three independent experiments. Two-tailed Student’s t test was used. *P < 0.05, **P < 0.01 and ***P < 0.001 and n.s. stands for no significance.

**Additional file 8: Figure S7. (A-C)** The mRNA expression of ACTL6A and CDK2, the protein expression of MYC in public human breast cancer datasets from TCGA. Two-tailed Student’s t test was used. *P < 0.05 and ***P < 0.001.

**Additional file 9: Figure S8. (A)** The percentage of high- or low-ACTL6A in non-TNBC and TNBC. **(B)** Kaplan-Meier Plotter program was used for analysis of RFS in TNBC and basal-like breast cancer patients groups. All settings were left at default values except the following ones: gene symbol (ACTL6A), survival (OS or RFS), and auto select best cutoff (on). **(C)** Multivariate cox regression analysis of RFS and OS for non-TNBC patients with low expression of ACTL6A versus high expression of ACTL6A.

## Data Availability

The data used and analyzed during the current study are available from the corresponding author on reasonable request.

## Abbreviations

ACTL6A: Actin-like protein 6A
TNBC: Triple-negative breast cancer
CDK: Cyclin-dependent kinase
HAT: Histone acetyltransferase
IP: Immunoprecipitation
GSEA: Gene Set Enrichment Analysis
ChIP: Chromatin immunoprecipitation
qPCR: Quantitative real-time PCR
IHC: Immunohistochemistry
TCGA: The Cancer Genome Atlas
PTX: Paclitaxel
RFS: Relapse-free survival
OS: Overall survival
