# Supplementary material for: Actin-like protein 6A/MYC/CDK2 axis confers high proliferative activity in triple-negative breast cancer
Source: J Exp Clin Cancer Res. 2021 Feb 4;40:56. doi: 10.1186/s13046-021-01856-3 (PMC7863242; doi:10.1186/s13046-021-01856-3)
Supplement: Supplementary file 1 — Additional file 1: Table S1. Primers. Table S2. Clinicopathological characteristics of 344 breast cancer patients and 220 triple-negative breast cancer patients. Table S3. Correlation between ACTL6A and clinicopathological characteristics of breast cancer patients. Table S4. Univariate and multivariate analysis of factors associated with 5-year overall survival and relapse-free survival in patients with TNBC. [file 13046_2021_1856_MOESM1_ESM.docx]

**Supplementary Table S1**

**Primers**

| **Primers for real-time PCR** | | | **Sequence(5'-3')** | |  |  |
| --- | --- | --- | --- | --- | --- | --- |
| ACTL6A | forward | | GGTTATGCTGGTGAGGACTGCC | |  |  |
|  | reverse | | TAGTAGGTGGGACCGCCTTGTT | |  |  |
| CDK2 | forward | | ATGGATGCCTCTGCTCTCACTG | |  |  |
|  | reverse | | CCCGATGAGAATGGCAGAAAGC | |  |  |
| GAPDH | forward | | GTCTCCTCTGACTTCAACAGCG | |  |  |
|  | reverse | | ACCACCCTGTTGCTGTAGCCAA | |  |  |
| CDK4 | forward  reverse | | CCATCAGCACAGTTCGTGAGGT  TCAGTTCGGGATGTGGCACAGA | |  |  |
| PCNA | forward  reverse | | CAAGTAATGTCGATAAAGAGGAGG  GTGTCACCGTTGAAGAGAGTGG | |  |  |
| RANBP1 | forward  reverse | | ACCATGACCCTCAGTTTGAGCC  AGTGCCTCGCTCCTTCCATTCT | |  |  |
| TYMS | forward  reverse | | GGTGTTTTGGAGGAGTTGCTGTG  GGAGAATCCCAGGCTGTCCAAA | |  |  |
| CCNA2  PSMA1  MAD2L1  PSMC4  PSMD14  PSMA4  TFDP1  UBE2E1  NOLC1 | forward  reverse  forward  reverse  forward  reverse  forward  reverse  forward  reverse  forward  reverse  forward  reverse  forward  reverse  forward  reverse | | CTCTACACAGTCACGGGACAAAG  CTGTGGTGCTTTGAGGTAGGTC  TGCAGAGCCATGTCCATTGGAG  GTAGTCAGGTCCTGTTCTGCAG  TTGAGTGTGACAAGACTGCAAAAG  CAGTGGCAGAAATGTCACCGTAG  GCAAGATGAACCTCTCTGAGGAG  CGGTTTTCACGGACAGCCAACA  GTCAGTGTGGAGGCAGTTGATC  CCACACCAGAAAGCCAACAACC  CTTGTGAGCAGTTGGTTACAGCG  AGCCATAGTGCTTATCCCAGCC  CACTTTGCCTCTCAGAACCAGC  CTTTCCTCTGCACCTTCTCGCA  CCTCCTTTCTATCTGCTCACTTC  GTAGCGTATCTCTTGGTCCACTG  GTAGCAGTGATGACTCAGAGGAG  CTGGAGGAATCCTCACTGCTAG | |  |  |
| PSMD7  PSMA6 | forward  reverse  forward  reverse | | GATGTGAAGCCGAAGGACCTAG  TCCTCAGCTTCCTCTGCTCCAA  GGATTCCAGCACAGTGACTCAC  ATGTCCACAGGAATCTCATAGCC | |  |  |
| **Primers for plasmid constructs** | | | | | | |
| pLVX-IRES-Hyg –ACTL6A | forward | | GgattcGCCACCATGAGCGGCGGCGTGTACGG | | | |
|  | reverse | | AgatctTCAAGGGCATTTTCTTTCTACACAC | | | |
| pMSCV-puro-retro  –MYC | forward | | GgattcCTGGATTTTTTTCGGGTAGTGGAAA | | | |
|  | reverse | | GaattcCCGAAGGGAGAAGGGTGT | | | |
| **Target sequence**  pSuper-retro-neo  –ACTL6A-shRNA-1 |  | | CTGGGATAGTTTCCAAGCTAT | | | |
| pSuper-retro-neo –ACTL6A-shRNA-2 |  | | CACCTACTACATAGATACTAA | | |  |
| pSuper-retro-neo  -MYC - shRNA | |  | | GAGGAAGAAATCGATGTTGTTTC | | |
| **Primers for ChIP** | |  | |  | | |
| CDK2 (P2) | | forward | | ACTCGGTGGGAGGCGGCAACAT | | |
|  |  | reverse | | TCGGGATGGAACGCAGTA | | |
| CDK2 (P1) | | forward | | CCACCTGCTTCAGCCTCCCA | | |
|  |  | reverse | | TGGGAGGCTGAAGCAGGTGG | | |

**Supplementary Table S2**

**Clinicopathological characteristics of 344 breast cancer patients**

| Parameters | | | Number of cases (%) | |
| --- | --- | --- | --- | --- |
| **Age** | | |  | |
| < 45 | | | 123 (35.8) | |
| ≥ 45 | | | 221 (64.2) | |
| **Clinical stage** | | |  | |
| I-II | | | 237 (68.9) | |
| III-IV | | | 107 (31.1) | |
| **T classification** | | |  | |
| T1-2 | | | 296 (86.0) | |
| T3-4 | | | 48 (14.0) | |
| **N classification** | | |  | |
| N0-N1 | | | 255 (74.1) | |
| N2-N3 | | | 89 (25.9) | |
| **Histologic grade** | | |  | |
| G1-2 | | | 175 (50.9) | |
| G3 | | | 169 (49.1) | |
| **ER status** | | |  | |
| Negative | | | 240 (69.8) | |
| Positive | | | 104 (30.2) | |
| **PR status** | | |  | |
| Negative | | | 246 (71.5) | |
| Positive | | | 98 (28.5) | |
| **HER-2 status** | | |  | |
| Negative | 313 (91.0) | |  |  |
| Positive | | | 31 (9.0) | |
| **TNBC**  No | | | 124 (36.0) | |
| Yes | | | 220 (64.0) | |
| **Vital status**  **alive**  **dead**  **Relapse** | | | 278(80.8)  66(19.2) | |
| No | | | 295 (85.8) | |
| Yes | | | 49 (14.2) | |
| **ACTL6A expression** | | |  | |
| Low | | | 200 (58.1) | |
| High | | | 144 (41.9) | |
|  | | |  | |

**Clinicopathological characteristics of 220 triple-negative breast cancer patients**

| Parameters | Number of cases (%) |
| --- | --- |
| **Age** |  |
| < 45 | 80 (36.4) |
| ≥ 45 | 140 (63.6) |
| **Clinical stage** |  |
| I-II | 147 (66.8) |
| III-IV | 73 (33.2) |
| **T classification** |  |
| T1-2 | 187 (85.0) |
| T3-4 | 33 (15.0) |
| **N classification** |  |
| N0-N1 | 160 (72.7) |
| N2-N3 | 60 (27.3) |
| **Histologic grade** |  |
| G1-2 | 104 (47.3) |
| G3 | 116 (52.7) |
| **Vital status**  alive  dead  **Relapse** | 179 (81.4)  41(18.6) |
| No | 188 (85.5) |
| Yes | 32 (14.5) |
| **ACTL6A expression** |  |
| Low | 113 (51.4) |
| High | 107 (48.6) |

**Supplementary Table S3**

**Correlation between ACTL6A and clinicopathological characteristics of breast cancer patients**

|  | | | |
| --- | --- | --- | --- |
|  | **ACTL6A expression** | |  |
| Characteristics | Low,  no. cases | High,  no. cases | *P* values |
| **Age**  < 45  ≥ 45 | 40  73 | 40  67 | 0.76 |
| **T** **classification** |  |  |  |
| T1-2 | 106 | 81 | < 0.001 |
| T3-4 | 7 | 26 |  |
| **N classification** |  |  |  |
| N0-1 | 95 | 65 | < 0.001 |
| N2-3 | 18 | 42 |  |
| **Histologic grade** |  |  |  |
| G1-2 | 56 | 48 | 0.485 |
| G3 | 57 | 59 |  |
| **Ki67** |  |  |  |
| < 14% | 41 | 21 | 0.006 |
| ≥ 14% | 72 | 86 |  |
| **Vital status** |  |  |  |
| Dead | 9 | 32 | < 0.001 |
| Alive | 104 | 75 |  |

**Supplementary Table S4**

**Univariate and multivariate analysis of factors associated with 5-year overall survival in patients with TNBC**

| Characteristics | Univariate analysis | | | Multivariate analysis | |
| --- | --- | --- | --- | --- | --- |
|  | HR (95% CI) | *P* values | HR (95% CI) | | *P* values |
| **Age**  (≥45 vs <45) | 0.792 (0.425-1.474) | 0.461 | 0.867 (0.458-1.676) | | 0.690 |
| **Expression of ACTL6A**  (high vs low) | 6.643 (3.145-14.035) | < 0.001 | 4.002 (1.775-9.021) | | < 0.001 |
| **T classification**  (T3-4 vs T1-2) | 4.478 (2.388-8.397) | < 0.001 | 2.049 (1.031-4.072) | | 0.041 |
| **N classification**  (N2-3 vs N0-1) | 4.945 (2.633-9.290) | < 0.001 | 2.458 (1.231-4.910) | | 0.011 |
| **M classification**  (N2-3 vs N0-1) | 5.103 (1.808-14.405) | 0.002 | 1.076 (0.337-3.442) | | 0.901 |
| **Histologic grade** (G3 vs G1-2) | 1.071 (0.575-1.996) | 0.828 | 0.731 (0.375-1.426) | | 0.358 |
| **Ki67**  (≥14% vs <14%) | 3.704 (1.452-9.450) | 0.006 | 2.134 (0.816-5.581) | | 0.122 |

HR, hazard ratio; CI, confidence interval.

**Univariate and multivariate analysis of factors associated with 5-year relapse-free survival in patients with TNBC**

| Characteristics | Univariate analysis | | | Multivariate analysis | |
| --- | --- | --- | --- | --- | --- |
|  | HR (95% CI) | *P* values | HR (95% CI) | | *P* values |
| **Age**  (≥45 vs <45) | 0.763 (0.410-1.420) | 0.763 | 0.862 (0.458-1.620) | | 0.644 |
| **Expression of ACTL6A**  (high vs low) | 6.252 (2.959-13.210) | < 0.001 | 3.692 (1.652-8.252) | | < 0.001 |
| **T classification**  (T3-4 vs T1-2) | 4.359 (2.325-8.172) | < 0.001 | 1.951 (0.988-3.851) | | 0.054 |
| **N classification**  (N2-3 vs N0-1) | 5.265 (2.802-9.892) | < 0.001 | 2.892 (1.476-5.666) | | 0.002 |
| **Histologic grade** (G3 vs G1-2) | 1.076 (0.577-2.007) | 0.819 | 0.763 (0.397-1.464) | | 0.415 |
| **Ki67**  (≥14% vs <14%) | 3.547 (1.390-9.052) | 0.008 | 1.913 (0.727-5.033) | | 0.189 |

HR, hazard ratio; CI, confidence interval.
