# Supplementary figures and images for: Actin-like protein 6A/MYC/CDK2 axis confers high proliferative activity in triple-negative breast cancer
Source: J Exp Clin Cancer Res. 2021 Feb 4;40:56. doi: 10.1186/s13046-021-01856-3 (PMC7863242; doi:10.1186/s13046-021-01856-3)

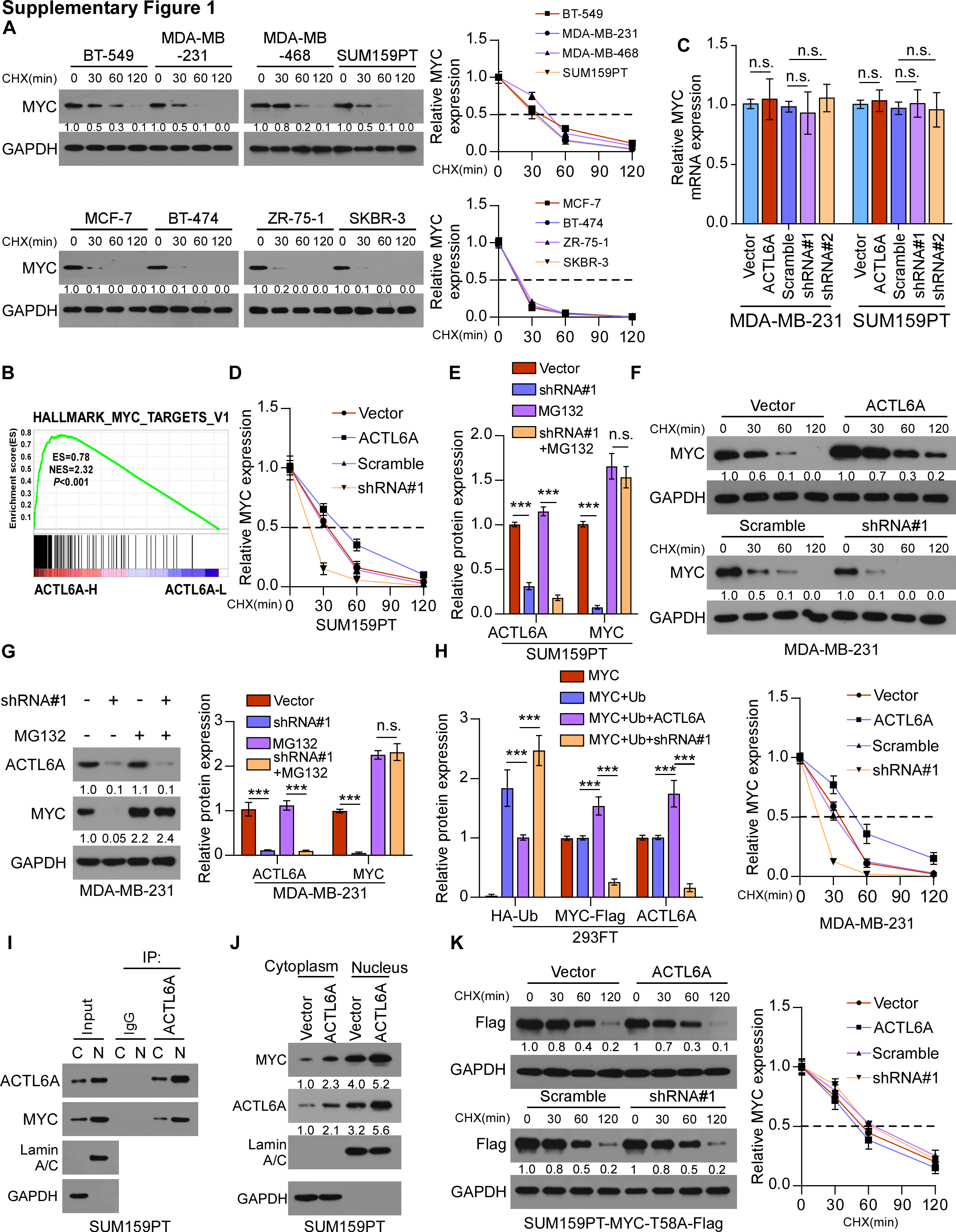

Supplement: Supplementary file 2 — Additional file 2: Figure S1. (A) Western blotting analyzed the MYC expression in 4 TNBC and 4 non-TNBC cell lines treated with CHX (50 μg/mL) for 0, 30, 60, or 120 min. GAPDH was used as loading control. The right panel was the statistical analysis. The MYC half-life’s was about 37 min in BT-549, 34 min in MDA-MB-231, 45 min in MDA-MB-468, 32 min in SUM159PT, 19 min in MCF-7, 19 min in BT-474, 20 min in ZR-75-1, and 20 min in SKBR-3. (B) Gene set enrichment analysis (GSEA) of TCGA dataset showed significant enrichment of MYC signature (HALLMARK_MYC_TARGETS_V1), in samples with high expression of ACTL6A. NES, normalized enrichment score. (C) Real-time PCR analysis of MYC in control, ACTL6A-overexpressed and -knockdown SUM159PT cells. (D) The statistical graph of Fig. 1e. The MYC half-life’s was about 45 min in ACTL6A-overexpressing group, 34 min in vector, 31 min in scramble groups, and 17 min in shACTL6A group. (E) The statistical graph of Fig. 1f. (F) Western blotting analyzed the MYC expression in MDA-MB-231 cells treated with CHX (50 μg/mL) for 0, 30, 60, or 120 min. GAPDH was used as loading control. The lower panel was the statistical analysis. The MYC half-life was about 50 min in ACTL6A-overexpressing group, 36 min in vector group, 31 min in scramble groups, and 14 min in shACTL6A group. (G) MYC protein level in the indicated cells under MG132 (10 μM) treatment for 8 h and then western blotting was conducted. GAPDH was used as loading control. (H) The statistical graph of Fig. 1g. (I) Subcellular IP assays were performed using anti-ACTL6A antibody in SUM159PT cells. C, cytoplasm; N, nucleus. (J) Immunoblot for the indicated proteins of subcellular fractions in SUM159PT cells with or without ACTL6A overexpression. Lamin A/C and GAPDH expression were used as nuclear and cytoplasmatic controls, respectively. (K) Western blotting analyzed the MYC expression in SUM159PT-MYC-T58A-Flag cell lines treated with CHX (50 μg/mL) for 0, 30, 60, or 120 min. GAPDH was used [file 13046_2021_1856_MOESM2_ESM.tif]

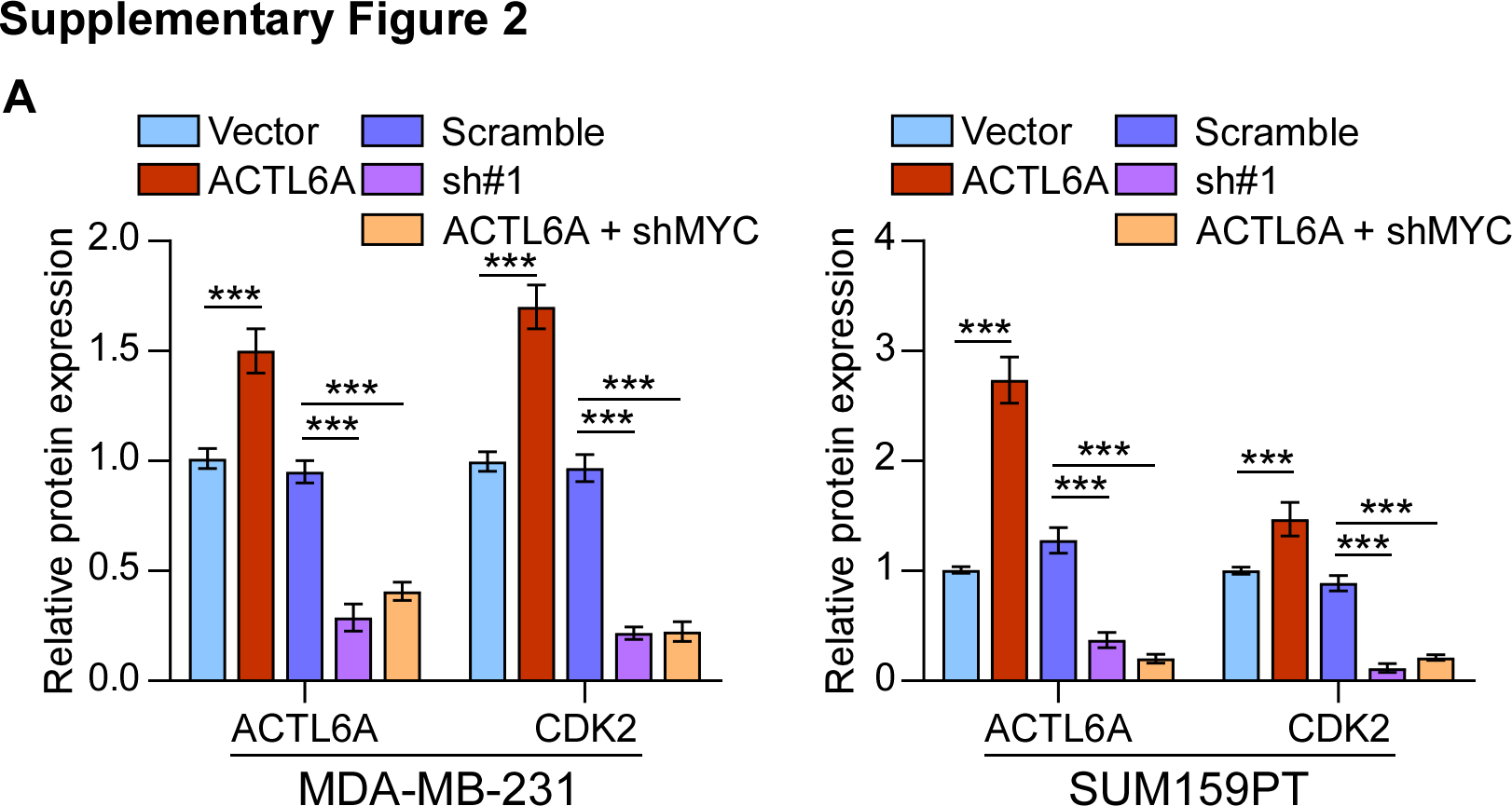

Supplement: Supplementary file 3 — Additional file 3: Figure S2. (A) The statistical graph of Fig. 2c. ***P < 0.001 [file 13046_2021_1856_MOESM3_ESM.tif]

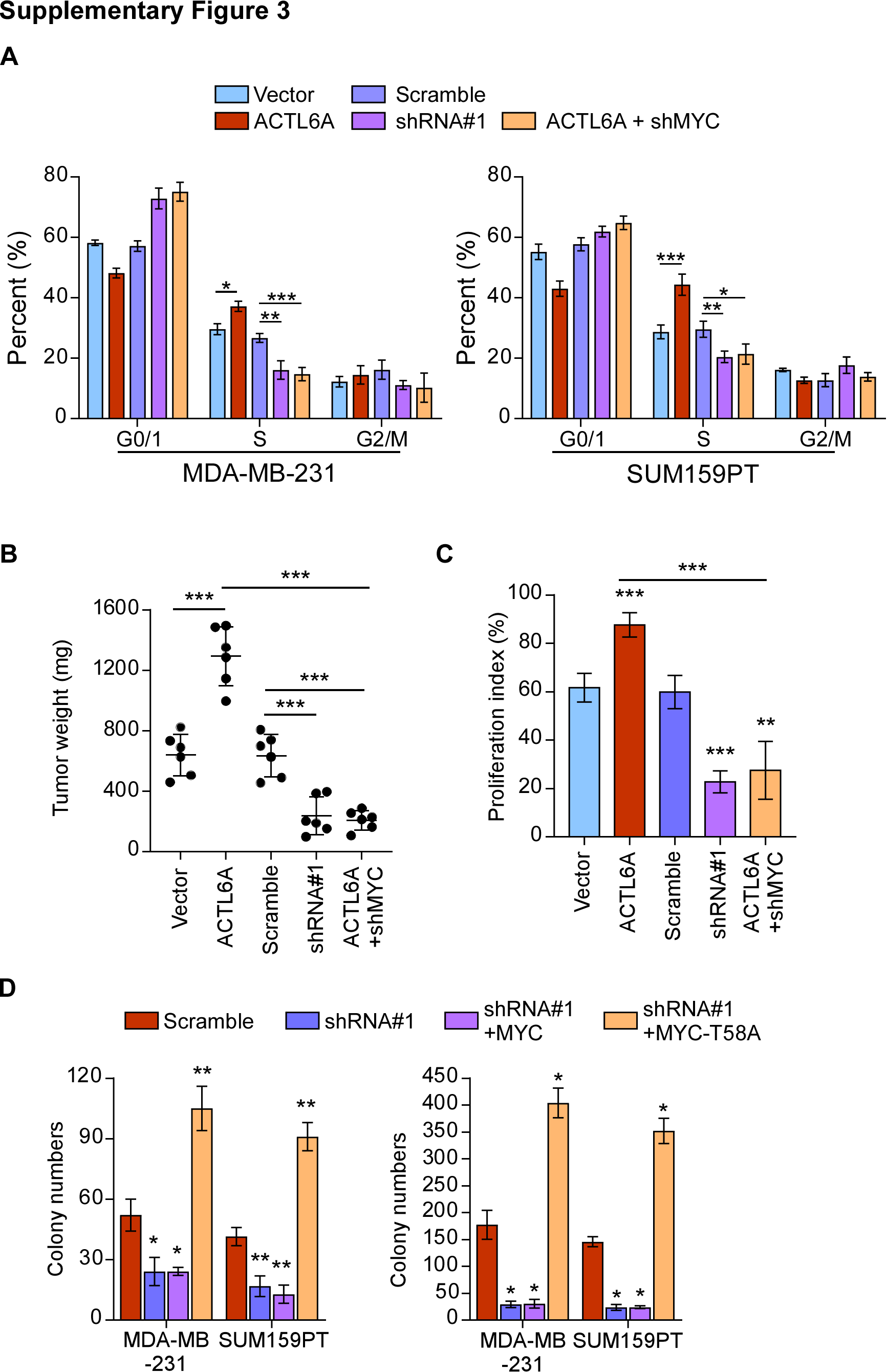

Supplement: Supplementary file 4 — Additional file 4: Figure S3. (A) The percentage of the indicated cells in G0/G1, S and G2/M phases was analyzed in flow cytometric analysis. (B) The tumor weights in the indicated groups are measured. (C) Percentage of Ki-67 were shown in the indicated cells. (D) Quantification of anchorage-independent growth colony formation and colony formation for the indicated cell lines. Two-tailed Student’s t test was used. *P < 0.05, **P < 0.01 and ***P < 0.001. [file 13046_2021_1856_MOESM4_ESM.tif]

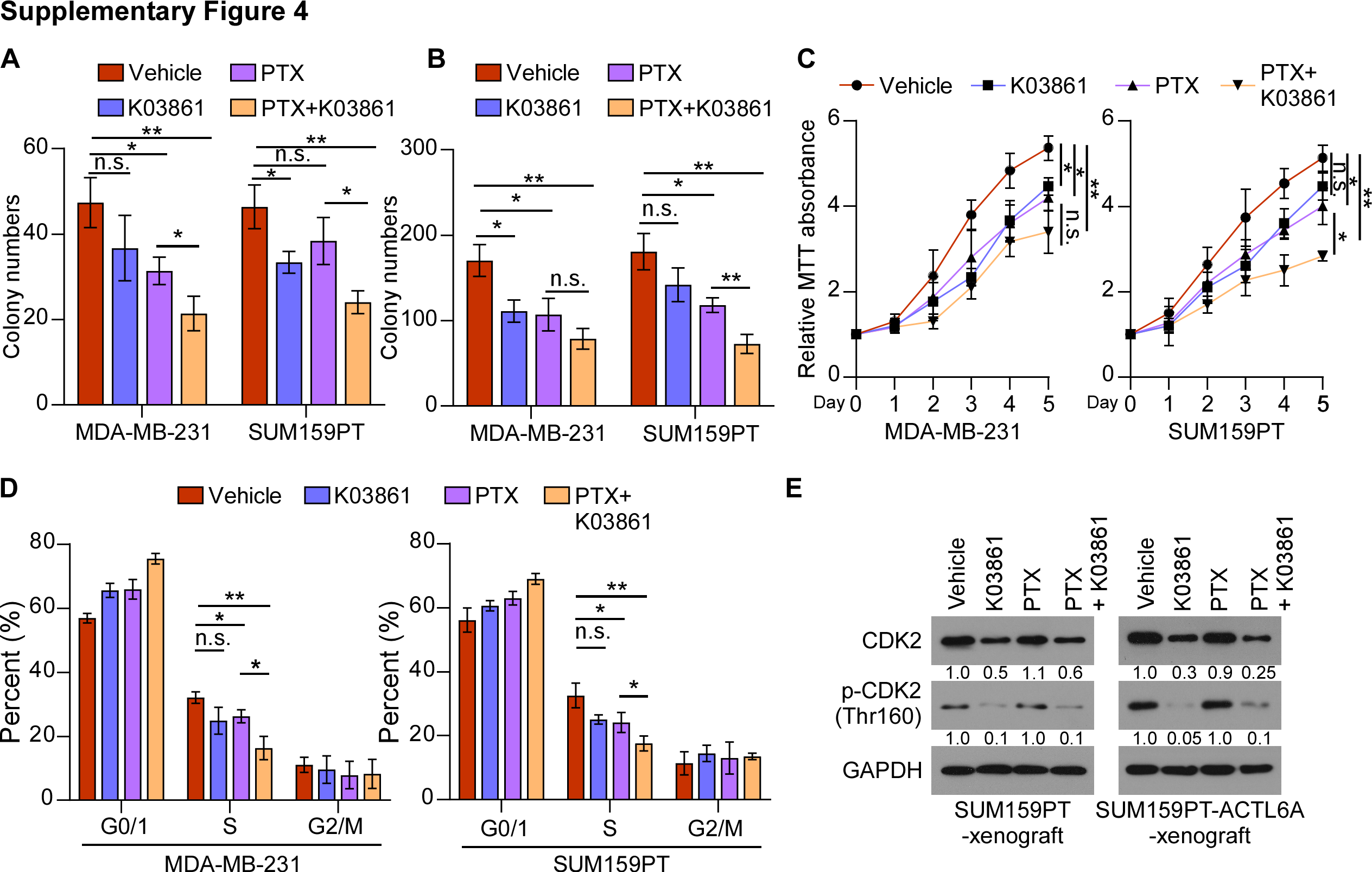

Supplement: Supplementary file 5 — Additional file 5: Figure S4. (A-C) Soft agar assay, colony formation assay and MTT assay were performed in vehicle, K03861 (50 nM), PTX (100 nM) or combination of K03861 and PTX groups in TNBC cells. (D) Flow cytometric analysis of the indicated cells with different treatments. (E) Immunoblot for the CDK2 and p-CDK2 (Thr160) proteins from SUM159PT and SUM159PT-ACTL6A tumors. GAPDH was used as loading control. *P < 0.05, **P < 0.01 and n.s. stands for no significance. [file 13046_2021_1856_MOESM5_ESM.tif]

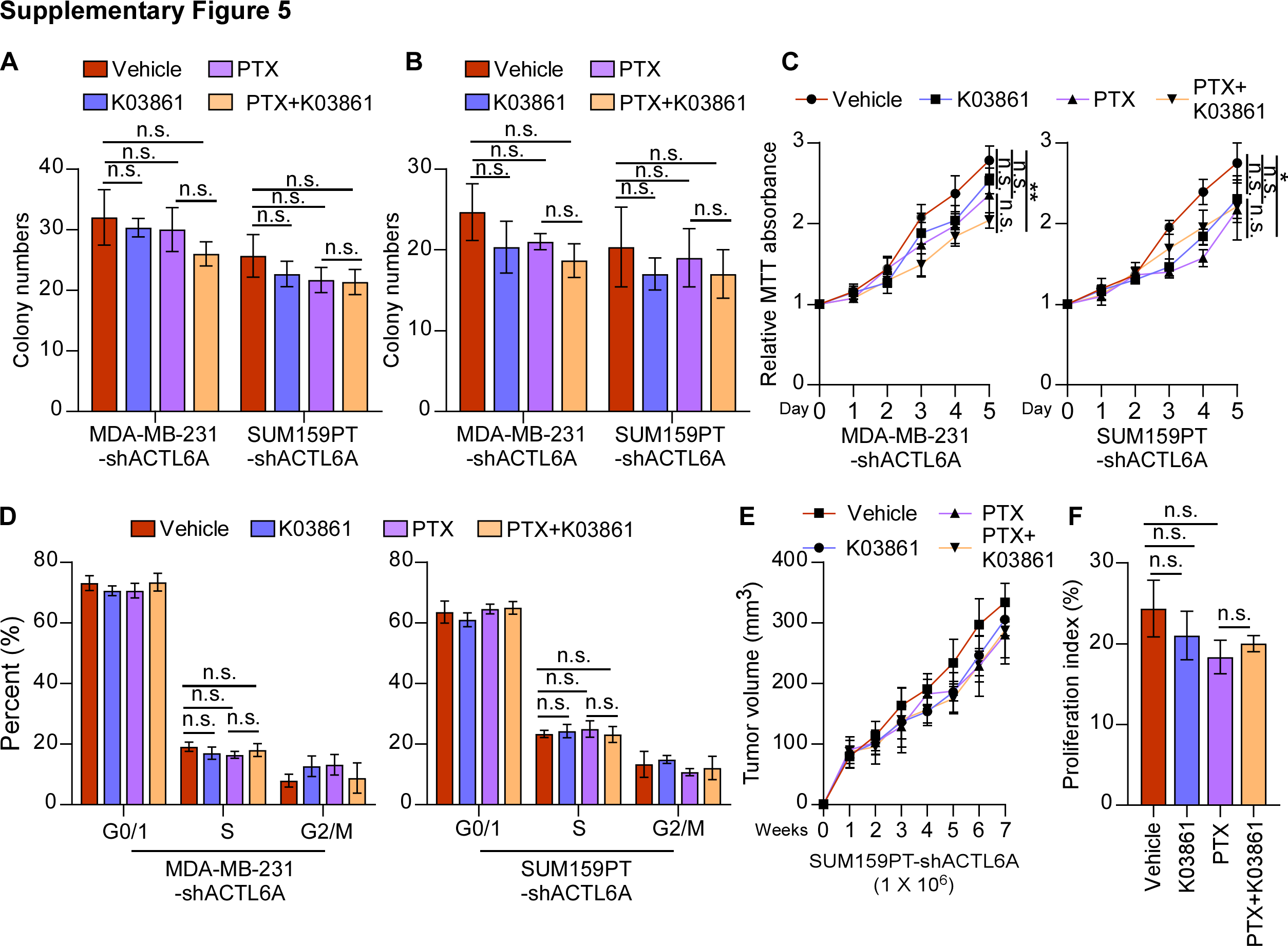

Supplement: Supplementary file 6 — Additional file 6: Figure S5. (A-C) Soft agar assay, colony formation assay and MTT assay were performed in vehicle, K03861 (50 nM), PTX (100 nM) or combination of K03861 and PTX groups in silencing ACTL6A cells. (D) Flow cytometric analysis of the indicated cells with different treatments. (E) ACTL6A-silenced cell lines SUM159PT were subcutaneously injected into mice (1 × 10^6^/injection, n = 6/group). One week after inoculation, the mice were intraperitoneally injected with K03861 (5 mg/kg, once a day for 5 consecutive days each week, for up to 7 weeks), PTX (10 mg/kg, once a day for 5 consecutive days each week, for up to 7 weeks), combination with two drugs or vehicle. The tumor volumes in each group are shown. (F) IHC of Ki-67 staining showed in the indicated xenografts. Data represent the means ± S.D. of three independent experiments. Two-tailed Student’s t test was used. *P < 0.05, **P < 0.01 and n.s. stands for no significance. [file 13046_2021_1856_MOESM6_ESM.tif]

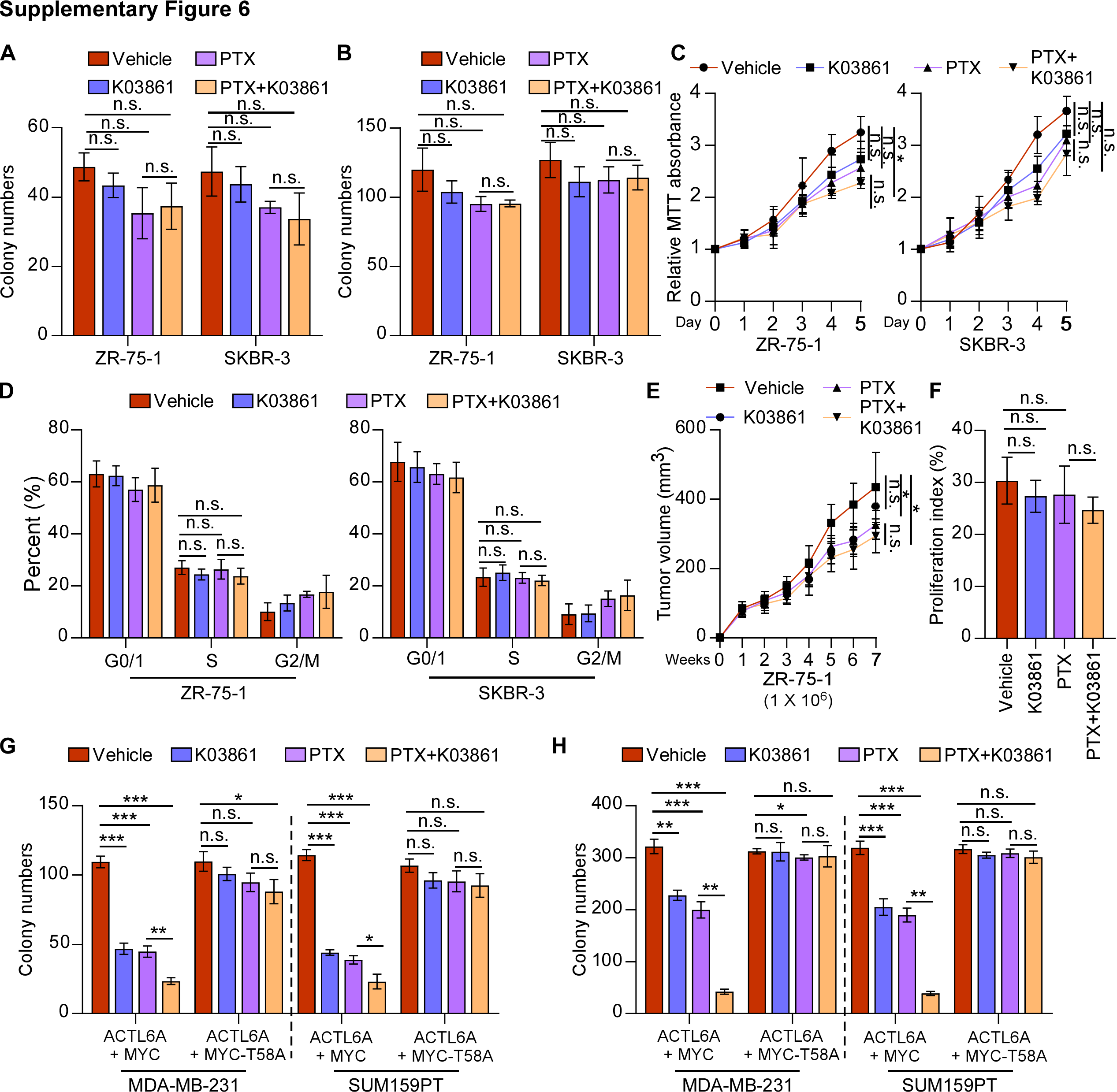

Supplement: Supplementary file 7 — Additional file 7: Figure S6. (A-C) Soft agar assay, colony formation assay and MTT assay were performed in ZR-75-1 and SKBR-3 cells treated with vehicle, K03861 (50 nM), PTX (100 nM) or combination with two drugs. (D) Flow cytometric analysis of the indicated cells with different treatments. (E) ZR-75-1 cells were subcutaneously injected into mice (1 × 10^6^/injection, n = 6/group). One week after inoculation, the mice were intraperitoneally injected with K0386, PTX, combination with two drugs or vehicle. The tumor volumes in each group are shown. (F) IHC of Ki-67 staining showed in the indicated xenografts. (G-H) Quantification of anchorage-independent growth colony formation (G) and colony formation (H) for the indicated cell lines. Data represent the means ± S.D. of three independent experiments. Two-tailed Student’s t test was used. *P < 0.05, **P < 0.01 and ***P < 0.001 and n.s. stands for no significance. [file 13046_2021_1856_MOESM7_ESM.tif]

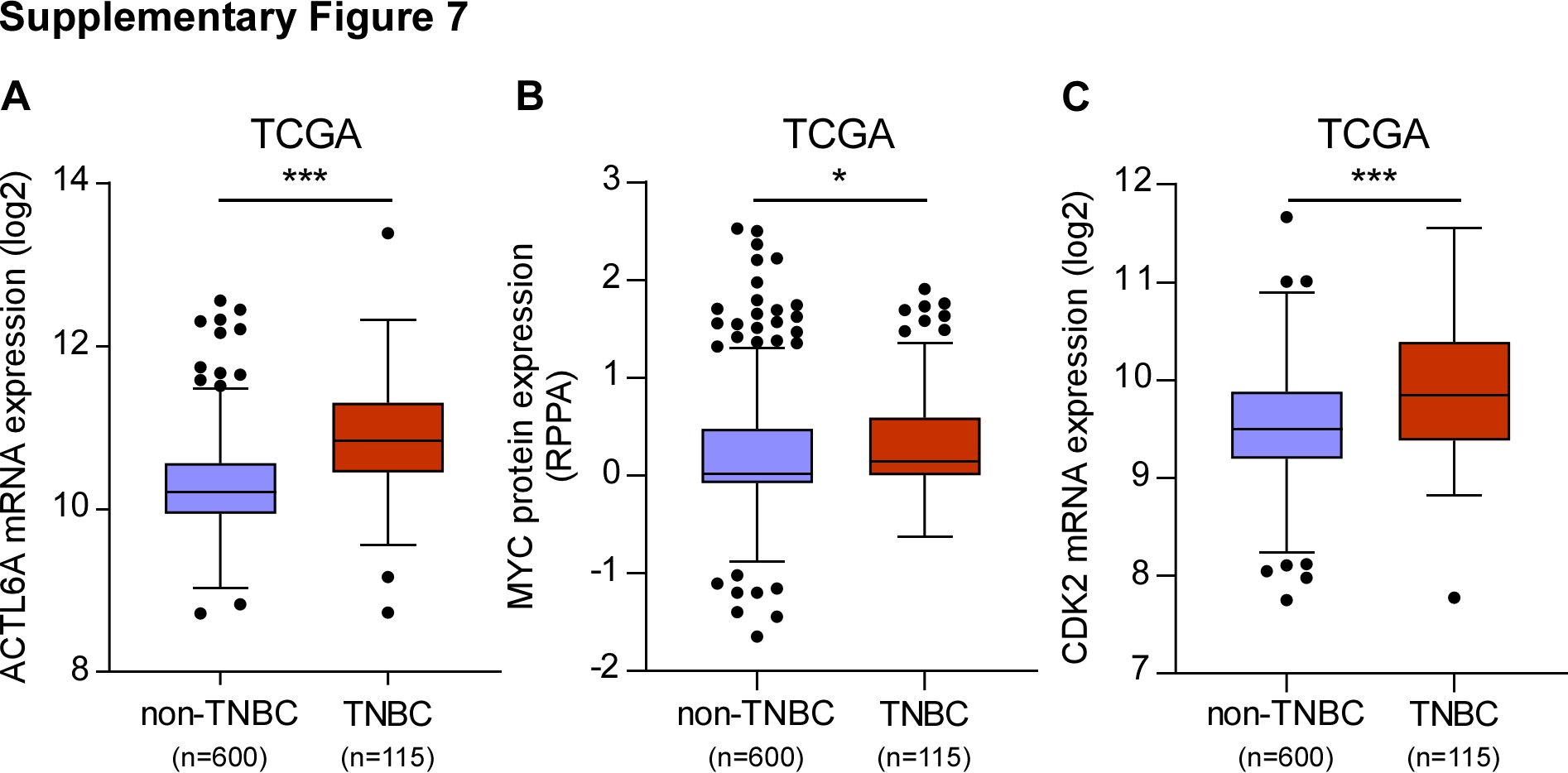

Supplement: Supplementary file 8 — Additional file 8: Figure S7. (A-C) The mRNA expression of ACTL6A and CDK2, the protein expression of MYC in public human breast cancer datasets from TCGA. Two-tailed Student’s t test was used. *P < 0.05 and ***P < 0.001. [file 13046_2021_1856_MOESM8_ESM.tif]

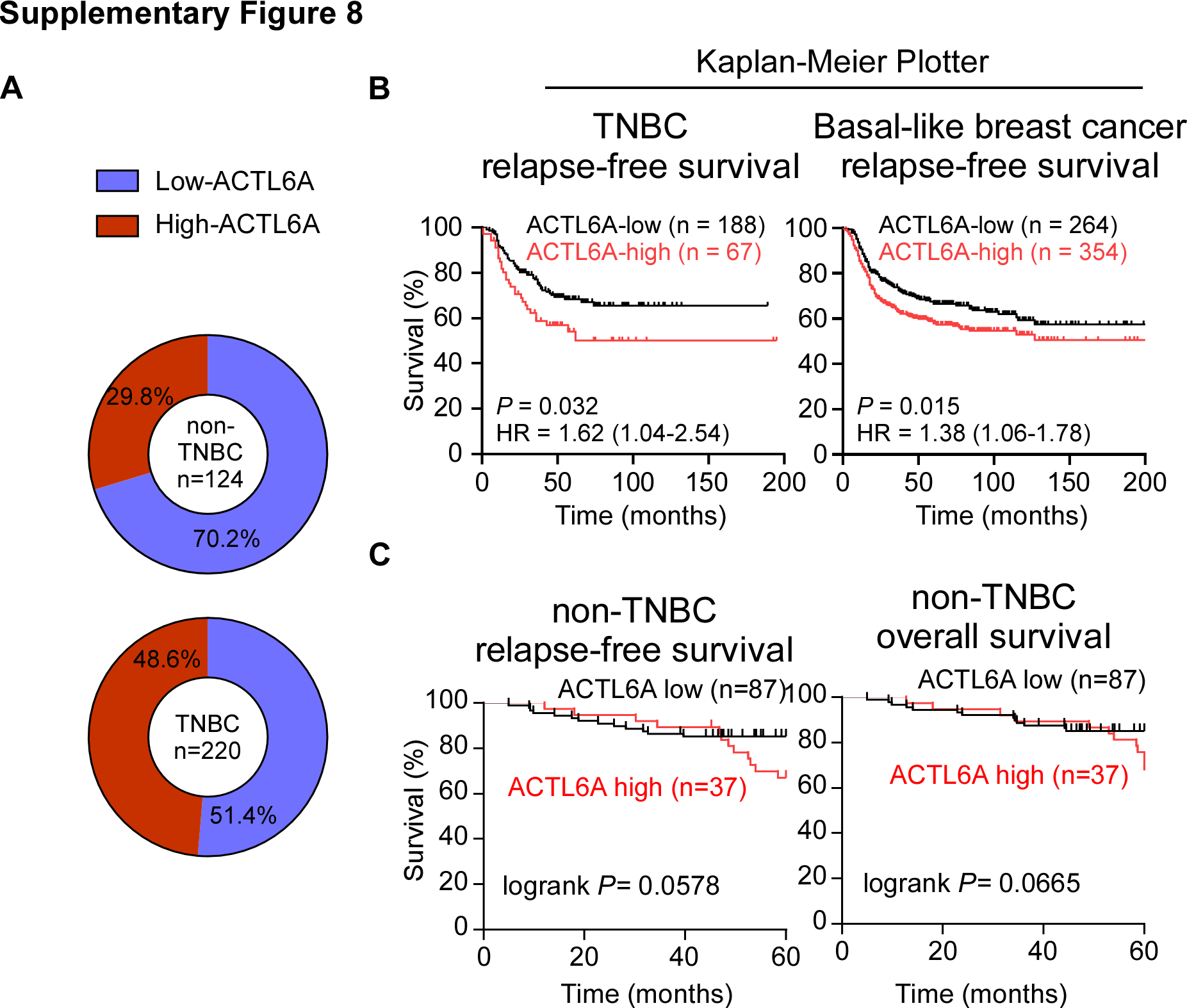

Supplement: Supplementary file 9 — Additional file 9: Figure S8. (A) The percentage of high- or low-ACTL6A in non-TNBC and TNBC. (B) Kaplan-Meier Plotter program was used for analysis of RFS in TNBC and basal-like breast cancer patients groups. All settings were left at default values except the following ones: gene symbol (ACTL6A), survival (OS or RFS), and auto select best cutoff (on). (C) Multivariate cox regression analysis of RFS and OS for non-TNBC patients with low expression of ACTL6A versus high expression of ACTL6A. [file 13046_2021_1856_MOESM9_ESM.tif]
